# Speed and current harmonics reduction using an adaptive proportional integral resonant controller for PMSM based electric vehicle drives

**DOI:** 10.1038/s41598-025-09981-1

**Published:** 2025-07-22

**Authors:** Elango Sangeetha, Vijaya Priya Ramachandran

**Affiliations:** 1 School of Electrical Engineering, Vellore Institute of Technology, Vellore, 632014 Tamil Nadu India; 2 School of Electrical Engineering, Vellore Institute of Technology, Chennai, 600127 Tamil Nadu India

**Keywords:** Electric vehicle, Permanent magnet synchronous machine, Proportional integral controller, Proportional integral resonant controller, Electrical and electronic engineering, Batteries

## Abstract

The use of permanent magnet synchronous machine (PMSM) in vehicle propulsion systems is growing in prominence. The machines provide greater torque density and efficiency as a result of PMSM pre-excitation. However, because of their poor torsional vibration dampening, their intrinsic torque ripple may provide a challenge for electric vehicles (EVs) and degrade passenger comfort. This may prohibit the utilization of PMSM to increase the energy economy of vehicles. This paper proposed a speed-current adaptive proportional-integral-resonant (PIR) control strategy to reduce periodic torque harmonics and provide smooth speed control of the PMSM drive system. The effects of several non-ideal components on speed and current components are analyzed according to their location in the system. The components include rotor flux harmonics, cogging torque, inaccurate current measurement including offset error and scaling error, and inverter dead time error, these components all lead to periodic torque harmonics. In order to determine the best phase adjustment parameters for the resonant item to minimize speed-torque harmonics and ensure system stability, stability analysis is carried out to take into account the delays brought on by the current loop and speed loop. Consequently, the PMSM drive system’s stability, overall performance, and efficiency are enhanced due to the decreased harmonics in the speed and current loop. Ultimately, the findings of the simulation and real-time simulator utilizing the OPAL-RT OP5700 platform show that the proposed adaptive PIR control method successfully lowers the periodic speed-current harmonics THD values of the PMSM drive when compared to conventional control strategies. The proposed control system is more stable and efficient as a result of lower THD values.

## Introduction

In the 2019 study, the United States environmental protection agency found that the transportation sector is responsible for 29% of the greatest percentage of greenhouse gas emissions. Additionally, carbon dioxide and other greenhouse gas emissions from the transportation sector are comparable in Canada, the United Kingdom, and France^1^. There is no denying that these pollutants have a detrimental effect on air quality and contribute to global warming. Most nations have made plans to move forward with transportation electrification using electric vehicles (EVs), which is an encouraging strategy. In Canada, for instance, the goal is for all newly sold light passenger cars to be EVs by 2035. To accomplish this goal, a federal incentive package for EV buyers has been approved, which includes a $2500 to $5000 incentive. EVs are, of course, the vehicle of the next generation^2^.

EVs have been equipped with a variety of electric machines (EMs) throughout the past few decades^3^. The most extensively utilized EM types in industry are induction motor (IMs) and asynchronous motors. Their benefits include their brushless design, adaptability to a wide range of conditions, straightforward construction, lack of commutators, and strong starting torque. However, their efficiency and power density are inferior, and their power factor is rather low, particularly when minimal mechanical load is applied. Other motors that have been used in EVs are switch reluctance motors (SRMs)^4–6^. SRMs are strong, durable, and appropriate for hard circumstances; they don’t have permanent magnets. SRMs perform exceptionally well in terms of fault tolerance. Nevertheless, they have several significant drawbacks: a higher acoustic noise level, a poorer power factor, and the requirement for a customized and specialized power inverter. Furthermore, they don’t provide good power density and need more input current to produce a given torque.

In the above literature, numerous types of EMs have been used for EVs. The most popular motors for EV applications are permanent magnet synchronous motors (PMSM)^7^ because of power density and efficiency of PMSMs are extremely high. It is also able to sustain their high efficiency across a broad spectrum of speed-torque characteristics. Moreover, it can generate less vibration and acoustic noise and run at steady power for the majority of their operating range. According to technical specifications, PMSM has a reluctant torque component that represents the permanent magnet’s influence in addition to its electromagnetic torque. Field weakening thus becomes a highly appreciated control technique for IPMSMs. Moreover, a range of control strategies have been employed to sustain the high operating level of the PMSMs^8^. However, the PMSM has intrinsic torque harmonics in many industrial applications. These torque harmonics cause periodic oscillations in the motor speed. These speed variations usually result in undesirable mechanical vibrations at low speeds and acoustic noise at high speeds, which also impair the efficiency of the servo system^9^. Periodic torque harmonics are typically caused by a variety of factors, including flux harmonics, cogging torque, phase unbalancing, and inaccuracies in current measurement.

Low frequency torque harmonics in PMSMs can create perceptible speed harmonics due to the rotational inertia of the motor not effectively filtering them. In low-inertia systems like direct-drive systems or light EV applications, this is particularly bothersome. Unlike high-frequency harmonics, which are often attenuated by the mechanical properties of the system, low-frequency harmonics can mirror the natural response of the mechanical system closely and create audible oscillations. Disregarding the load torque effect, torque harmonics mainly occur due to inefficient system components, for example, motor flux harmonics, cogging torque^10–15^, current measurement errors^16^, and dead time error of the inverter^17,18^. In an effort to minimize the speed harmonics, studies on motor design and control algorithm have been pursued. Whereas research on the latter seeks to accurately control motor speed with a range of feed-forward and/or feedback control strategies, the former focuses on optimal PMSM design and eliminates motor torque harmonics through skewed slots and poles. To compute reference current, feed-forward control needs offline pre-knowledge of torque harmonics, and the quality of this pre-knowledge decides how effectively it performs^19^. Conversely, feed-back control employs state variables such as torque or speed to formulate feed-back laws. Internal model concepts are usually recognized in the design of feedback controllers^20,21^. In^22^, a rule of speed feedback is formulated, and the speed controller utilizes the time-varying rotor flux model. Torque harmonics are effectively eliminated by adaptive model parameter estimation. An interior model of frequency-domain disturbances is constructed in^23,24^ with a variable-structure sliding mode controller, which although having robust dynamic performance, is plagued by unavoidable chattering. The torque harmonics due to non-ideal reasons can be considered periodic functions of the rotor position owing to the symmetrical structure and operation of PMSMs.

Periodical controllers are well suited due to PMSMs symmetric structure and functioning, which permits torque harmonics due to non-ideal origins to be thought of as being periodic functions with respect to rotor position. Speed harmonics amounts are studied in^25^ where a proportional integral (PI) approach in the speed loop combined with iterative learning is used efficiently to suppress harmonics at a variety of frequencies. In order to obtain zero steady-state error in tracking the periodic parts of the reference current, a repetitive current control method is suggested in^26^. Although iterative learning algorithm and repetitive controller algorithm provide accurate reference tracking, total disturbance rejection, and infinite gain over some frequency ranges, they also carry the risk of instability. This risk is especially evident in speed loops, where phase margins may go negative at high frequencies because of delays in the current loop and speed measurement.

Internal model principle-based control strategies like resonant and repetitive controllers are best methods to mitigate speed ripple. However, repetitive controllers have the limitation that they need sampled frequencies of variable values, making frequency adaptation cumbersome^27–30^. Resonant controllers allow precise reference tracking, rejection of disturbances, and immeasurable gain at the frequency of disturbance. They have been extensively applied in devices like active power filters, pulse width modulation rectifiers, and the like. In order to suppress unwanted peaks at certain frequencies, a harmonic reduction method based on resonant control with complex vector control has been suggested. For the analysis and design of proportional resonant (PR) controllers, the phase-margin criterion and bode diagrams are widely employed. But this technique is faced with constraints whenever resonant frequencies are higher than the crossover frequency specified by the proportional gain. In order to improve system stability, the effect of time delays on resonant control systems is demonstrated and relevant phase compensation methods are proposed in^31–34^.

Phase-compensated resonant controllers are widely used in grid-side applications but are rarely applied in motors for reducing speed ripple^35^. In grid-side applications, the resonant frequency is typically *6k ± 1* times the grid frequency, whereas in machine applications, it is constantly influenced by rotational speed fluctuations. Consequently, designing a phase-compensated resonant controller for PMSM drives requires addressing the significantly more complex issue of frequency adaptation compared to grid-side systems. To mitigate torque ripple caused by non-sinusoidal back electromotive force (EMF) in surface-mounted PMSMs, several resonant controllers with frequency-adaption prominent angle compensation have been implemented in the *q*- coordinate axis^36^. However, this approach is unsuitable in scenarios involving current measurement inaccuracies, where the resonant controller must instead be incorporated into the outer loop of the speed controller.

Important stability characteristics and conditions are discussed in^37^, which provides tuning techniques for predictive-integral resonant control in PMSM drives to minimize periodic disturbances caused by current sampling errors and dead time effects. However, the phase correction design for resonant controller, a critical factor for maintaining sufficient stability margins, particularly at high speeds, is not adequately addressed. To attain smooth speed control for low-speed PMSM drives, a proportional-integral-resonant (PIR) control strategy is recommended for both the speed and current loops^38^. However frequency adaptation is not considered. Furthermore, despite their criticality, stability restrictions at various speeds are not examined. If stability margins are not appropriately taken into account, incorporating a resonant controller can soon result in system instability, especially in the speed loop^39^.

The above issues are solved by proposing an adaptive PIR controller with phase compensation for speed and current ripple minimization in PMSM drive EV systems. The resonant controller of the speed-current loop is constructed in parallel with the PI controller. Classical control theory is applied to specify the parameters for the proposed PIR with phase compensation control system to enable resonant frequency adaptation and ensure system stability. If the resonant frequency is below the cut-off frequency of the original PI controller, then phase adjustment is not required since the only aspect that impacts stability in the resonant gain. If the resonant frequency is higher than the cut-off frequency, a piecewise compensation method is used in order to obtain the best stability margin, harmonic reduction capacity and improve the overall performance and efficiency of the system for every speed range of the PMSM drive. As a result, a sophisticated real-time mathematical model of the compensatory angular function may no longer be required. Based on an examination of the non-ideal components and their effects on the control system, this paper proposed a current and speed PIR control method that takes use of the periodic nature of torque and speed harmonics of PMSMs.

The main objectives of the proposed system summarized as follows.

(i) This study proposed a speed and current loop PIR controller to address the issue that arises mostly from conventional control methods because of an non-deal factors of PMSM drive system.
(ii) The proposed drive system is effectively implemented into an EV drive control system based on PMSM to enhance system stability and lower overshoot, settling time, and steady state error.
(iii) The torque and current harmonics are likewise reduced by the proposed system. Vibration should decrease and system efficiency should rise as a result of the harmonics being reduced.
(iv) MATLAB/Simulink and the OP5700 platform are used to confirm the dive system’s efficiency.

The rest of this paper is summarized as follows. Section II provides the detailed explanation of periodicity of the torque ripple it contains flux harmonics, cogging torque, current measurement error including offset and scaling error, effect of dead time on voltage source inverter (VSI). The speed and current control design process is investigated in Section III. Section IV investigates the simulation and experimental results verification, Section VI describes the discussion with the other control techniques. Finally, Section V concludes this article.

## Torque ripple periodicity

The mathematical representation of PMSM in synchronous rotating coordinates^37^, avoiding magnetic saturation, hysteresis losses, and eddy current losses, is as follows:

1 $$\:{v}_{sd}=\:{R}_{s}{i}_{sd}+\:{L}_{sd}\frac{{di}_{d}}{dt}-p\omega\:\:\left({L}_{sq}{i}_{sq}+{\phi\:}_{sqm}\right){v}_{sq}=\:{R}_{s}{i}_{sq}+\:{L}_{sq}\frac{{di}_{q}}{dt}-p\omega\:\:\left({L}_{sd}{i}_{sd}+{\phi\:}_{sdm}\right).$$

From the above equations $\:{v}_{sd}$ and $\:{v}_{sq}$ are the *d-* and *q-* axis stator voltage; $\:{i}_{sd}\:$and $\:{i}_{sq}$ are the *d* and *q* axis stator currents respectively; $\:{L}_{sd}$ and $\:{L}_{sq}$ are the *d-* and *q-* axis stator inductance, respectively; $\:{R}_{s}\:$is the stator resistance, $\:{T}_{e}\:$represents the electromagnetic torque, $\:{T}_{cog}$represents the cogging torque, $\:{T}_{L\:}$ represents the load torque, $\:\omega\:$ represents the mechanical angular speed,

2 $$\:J\frac{d\omega\:}{dt}=\:{T}_{e}-{T}_{cog}-\:{T}_{L\:}-B\omega\: .$$

From the above equation *B* is the friction coefficient, *p* represents the number of pole pairs, $\:J$ indicates the rotor moment of inertia, $\:{\phi\:}_{sdm}$ and $\:\:{\phi\:}_{sqm}$ represents the *d* and *q* axis permanent magnet fluxes, respectively.

The equation for electromagnetic torque is written as,

3 $$\:{T}_{e}=1.5p\:\left[\left({\phi\:}_{sdm}+\:{L}_{sd}{i}_{sd}\:\right){i}_{sq}-\left({\phi\:}_{sqm}+{L}_{sq}{i}_{sq}\right){i}_{sd}\right].$$

From the above equation $\:{T}_{e}$represents the electromagnetic torque, *p* represents the number of pole pairs, $\:{\phi\:}_{sdm}$ and $\:\:{\phi\:}_{sqm}$ represents the *d* and *q* axis permanent magnet fluxes, $\:{L}_{sd}$ and $\:{L}_{sq}$ are the *d-* and *q-* axis stator inductance, $\:{i}_{sd}\:$and $\:{i}_{sq}$ are the *d* and *q* axis stator currents respectively.

The Eq. (3) can be simplified using the $\:{i}_{sd}=0$ control method, hence it can be written as

4 $$\:{T}_{e}=1.5p{\phi\:}_{sdm}{i}_{sq}.$$

The simplified electromagnetic torque equation is represented in (4) the torque ripple is calculated using below Eq. (5)

5 $$\:{T}_{ripple}=\frac{{T}_{max}-{T}_{min}}{{T}_{avg}}.$$

From the above equation $\:{T}_{max}$ represents the maximum torque,$\:\:{T}_{min}$ is the minimum torque,$\:\:{T}_{avg}$ is the average torque of the PMSM drives.

**Fig. 1:**
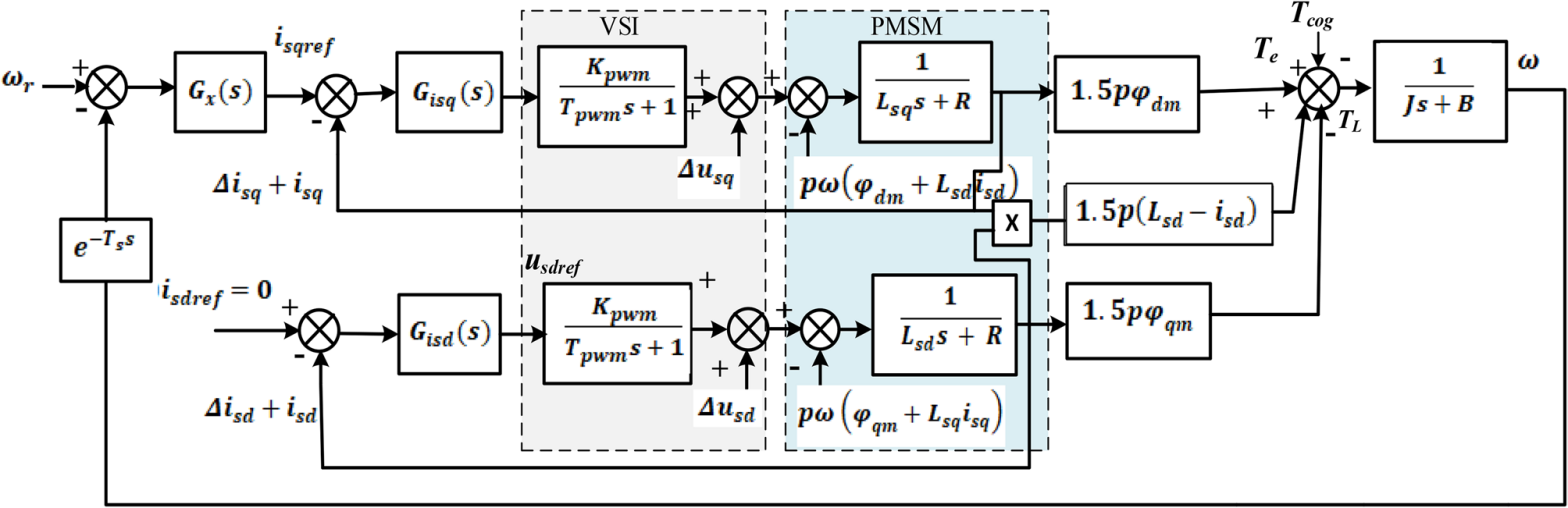
Block diagram of speed and current closed loop control.

The speed and current closed-loop control system is illustrated in Fig. 1 for PMSM based EVs. In Fig. 1$\:{\omega\:}_{r}$ represents the reference speed, $\:{i}_{sqref}\:$ represents the *q*-axis reference current and $\:{i}_{sdref}\:$ represents the *d-*axis reference current respectively and *G*_*x*_*(s)* representing the transfer functions of the speed controller. $\:{G}_{isd}\left(s\right)\:$and $\:{G}_{isq}\left(s\right)\:$representing the transfer functions of the *d*-axis and *q-*axis current controller, respectively. The inverter’s equivalent delay is$\:{\:T}_{pwm}$, and its corresponding gain is$\:{\:K}_{pwm}\:$. Where $\:{\varDelta\:i}_{sd}$ and $\:{\varDelta\:i}_{sq}$ are measurement errors as of right now. It can be mentioned that the actual machine drive system uses an optical encoder to obtain the data position, from which it then determines speed using the back differentiation approach. There will unavoidably be a delay in this process. As a result, the speed feedback channel gains a delay portion$\:\:{e}^{-{T}_{s}s}$, and the delay time $\:{T}_{s}$is determined by the computation method used.

### Harmonics of flux

When PMSM is an ideal mode, $\:{\phi\:}_{sqm}$ is considered as zero and when rotor flux orientation is used, $\:{\phi\:}_{sdm}$ only possesses a dc component. However, manufacturing constraints make it challenging to attain the optimal sinusoidal flux density distribution. In realistic PMSM, harmonics are often found in $\:{\phi\:}_{sqm}$ and$\:\:{\phi\:}_{sdm}$.

6 $$\begin{aligned}\:{\phi\:}_{sdm}&=\:{\phi\:}_{sd0}+\sum\:_{n=1}^{\infty\:}{\phi\:}_{sdn}\text{cos}\left(6{\theta\:}_{e}\right) \\ \:{\phi\:}_{sqm}&=\:\sum\:_{n=1}^{\infty\:}{\phi\:}_{sdn}\text{cos}\left(6{\theta\:}_{e}\right) \end{aligned}.$$

Where $\:{\phi\:}_{sd0}$represents the *d*-axis flux in the dc component, $\:{\phi\:}_{sdn}$and $\:{\phi\:}_{sqn}$ represent the amplitudes of the flux harmonic of ac component of the *d-* and *q-*axis and *n* is a positive integer.

As shown in Fig. 1, flux harmonics have a detrimental effect on speed and a current loop, which should result in periodic disturbances and lower PMSM drive performance. It should be evident from this picture that the flux components are utilized in feed-forward paths, which causes the PMSM drive to produce harmonics. As a result, the speed and current loop system performance are reduced. Flux harmonics have an impact on the production of electromagnetic torque $\:{T}_{e}$ in the speed loop and result in *6th* periodic variations in both torque and speed. Additionally, *6th* order components must be present in the speed controller *G*_*x*_*(s)* output owing to reduce speed harmonics. The back EMF of the current loop comprises the *6th* harmonics order and functions as a disruption. These harmonics will provide more stringent requirements for the current controller when combined with the ac components in the current reference.

### Cogging torque

Cogging torque is a periodic torque produced by the motor’s rotor and stator’s propensity to independently adapt to the position with the least amount of resistance. Consequently, even when the PM machine is not powered, cogging torque is present using finite element analysis method^40,41^. The cogging torque can be represented as a differential equation between the air gap and the magnetic energy *W* in the PMSM in relation to the rotor’s mechanical angle.

7 $$\:{T}_{cog}=-\:\frac{\partial\:W}{\partial\:{\theta\:}_{r}}.$$

From the above equation $\:{\theta\:}_{r}$ is the mechanical angle of the rotor position.

An integral of the magnetic energy density is one way to represent the magnetic energy.

8 $$\:W=\:{W}_{airgap+PMSM}=\:\frac{1}{2{\mu\:}_{a}}\int\:{D}^{2}dV.$$

From the above equation $\:{\mu\:}_{a}$ is the air permeability, *D* is the magnetic field density, and *V* is the volume of magnetic field.

The cogging torque can be represented as a Fourier series due to its periodicity as follows.

9 $$\:{T}_{cog}=\:\sum\:_{n=1}^{\infty\:}{T}_{n}\text{sin}\left(n{N}_{co}{\theta\:}_{e}/p\right).$$

Where $\:{T}_{n}$ represents the amplitude of the *nth* component of the Fourier series and $\:{N}_{co}$ is the least common multiple between motor slots and poles. In accordance with (6), it is shown in Fig. 1. Cogging torque causes periodic disturbances in the outer speed loop. Cogging torque is the primary cause of the torque ripple in the machine, and this study focuses on finding solutions for the low speed and low torque issue in EVs.

### Current measurement error

From the current measurement error, the dc offset error and the scaling error can be separated. dc offset inaccuracy is mainly caused by the intrinsic offset of the analogue sensors and the uneven dc supply voltage of the current sensors. If non-ideal effects from filters and converters are disregarded, the obtained current data can be expressed as,

10 $$\:{i}_{sm}=\:\frac{{R}_{s}}{{R}_{sN}}i+\frac{{u}_{o}-{u}_{o}N}{r{R}_{sN}}=Ki+{i}_{o}.$$

Where *r* is the current sensor’s transducer ratio and $\:{R}_{sN}$ and $\:{u}_{o}N$ are the nominal values for the offset voltage and sample resistance, respectively.

Equation (10) states that there are two primary elements that tend to alter the accuracy of current measurement. First, there is the difference between$\:{\:u}_{o}$and $\:{u}_{o}N$. Unbalanced positive and negative sensor feeding voltages as well as zero voltage drift in analogue devices are typically the causes of this deviation. The other is the fluctuating sampling resistance $\:{R}_{s}$ brought on by an increase in temperature. These two factors result in proportional and offset errors between $\:{i}_{sm}$and $\:i$. The errors in the dc offset is denoted by $\:{i}_{oa}$,$\:{i}_{ob}$ and proportional coefficients of the current in phases *a* and *b* are denoted by $\:{K}_{a}$,$\:{K}_{b}$ respectively; When the examined values for $\:{i}_{a}$and $\:{i}_{b}\:$are defined as $\:{i}_{ma}$ and $\:{i}_{mb}$ then,

11 $$\begin{aligned}\:{i}_{ma} &=\:\:{K}_{a}\:{i}_{a}+\:{i}_{oa}\:\\ \:{i}_{mb} &=\:\:{K}_{b}\:{i}_{b}+\:{i}_{ob}\: \end{aligned}.$$

Using synchronous rotating coordinates, convert (10) and assume that $\:{K}_{a}$=$\:\:{K}_{b}$, = 1.

12 $$\begin{aligned} \:{\varDelta\:i}_{sd}\:& =\:\frac{2}{\sqrt{3}}\:\:\sqrt{{i}_{oa}^{2}+{i}_{oa}{i}_{ob}+{i}_{ob}^{2}}\:sin\:\left({\theta\:}_{e}+\alpha\:\right) \\ \:{\varDelta\:i}_{sq}\:& =\:\frac{2}{\sqrt{3}}\:\:\sqrt{{i}_{oa}^{2}+{i}_{oa}{i}_{ob}+{i}_{ob}^{2}}\:cos\:\left({\theta\:}_{e}+\alpha\:\right) \end{aligned}.$$

From the above equation $\:{\varDelta\:i}_{sq}$ = *i*_*mq*_ − $\:{i}_{sq}$; $\:{\varDelta\:i}_{sd}\:$ = *i*_*md*_ − $\:{i}_{sd}\:$ ; where $\:{i}_{sd}$ and $\:{i}_{sq}$are the *d*- and *q*-axes real current values respectively; The measurements for $\:{i}_{sd}$ and $\:{i}_{sq}$ are denoted by *i*_*mq*_ and *i*_*md*_, respectively; where α is the angle that separates the rotor location from the stator current vector.

When $\:{i}_{oa}$ = $\:{i}_{ob}$ = 0, the result is

13 $$\begin{aligned} \:{\varDelta\:i}_{sd}\: &=\:\frac{\sqrt{3}}{3}\:\:\left({K}_{b}-\:{K}_{a}\right)I\:sin\:\left(2{\theta\:}_{e}+\beta\:\right)+\frac{2-{K}_{a}-\:{K}_{b}}{2}\:I \\ \:{\varDelta\:i}_{sq}\: &=\:\frac{\sqrt{3}}{3}\:\:\left({K}_{b}-\:{K}_{a}\right)I\text{sin}\left(2{\theta\:}_{e}+\beta\:\right)-\frac{\sqrt{3}}{6}\:\:I\left({K}_{a}-\:{K}_{b}\right)\:I \end{aligned}.$$

As per the equation above, *I* represent the phase current amplitude, and *β* is the angle created by the stator current vector and the rotor position. The current feedback channels will be perturbed by current measurement errors, as evident from (12), (13), and Fig. 1. Both the fundamental frequency and the frequencies present in the disturbances are double and the same. It must be noted that the above disturbances are hard to remove by using the present controller although the transfer functions from $\:{\varDelta\:i}_{sd}$ ($\:{\varDelta\:i}_{sq}$) to $\:{i}_{sd}\:$($\:{i}_{sq}$) and $\:{i}_{sdref}$ ($\:{i}_{sqref}$) to $\:{i}_{sd}$ ($\:{i}_{sq}$) are identical. One approach is to move $\:{\varDelta\:i}_{sq}$ to the beginning of current loops. Because it is considered an interference of the speed loop, the speed controller may then remove$\:{\:\varDelta\:i}_{sq}$.

### Effect of dead time on VSI

Dead time effect essential be added to the drive signals of the higher and lower legs of each phase of a PWM type VSI in order to prevent a dc link short circuit^42^. When considering space vector PWM symmetrical modulation, for instance, $\:{\varDelta\:u}_{a}$the discrepancy between phase a’s reference and actual voltages, can be roughly represented as

14 $$\:{\varDelta\:u}_{sa}\approx\:\:\left[\frac{{T}_{d}+\:{T}_{on}-\:{T}_{off}}{{T}_{s}}\:\left({U}_{dc}-\:{U}_{sat}+\:{U}_{f}\right)+\frac{{U}_{sat}+\:{U}_{f}}{2}\right]\:sgn\:\left({i}_{sa}\right).$$

The insulated-gate bipolar transistor’s (IGBT) dead time, turn-on, and turn-off delays are denoted by $\:{T}_{d}$, $\:{T}_{on}$, and $\:{T}_{off}$, respectively; $\:{T}_{s}$ is the PWM control cycle; dc link voltage represented by $\:{U}_{dc}$; $\:{U}_{sat}$ is the IGBT conduction voltage drop; $\:{U}_{f}$ is the conduction voltage drop of diode, respectively. Similar definitions of $\:{\varDelta\:u}_{b}$ and $\:{\varDelta\:u}_{c}$ for phases b and c may be found in (14).

As a result, the motor experiences torque and speed harmonics. In addition to affecting the reference current, nonideal elements in the drive system also cause dc and sporadic disturbances in the current loop and the speed loop.

## Design of the controller

The internal model principle states that in order to accurately track or completely suppress a periodic disturbance or periodic reference, the controller must build an internal model of it. Periodic signals can be represented by trigonometric series^43,44^. Therefore, as with cosine signals, the internal model of a periodic signal can be constructed from a series of resonant terms. In this study, a PI controller is connected in parallel with multiple resonant terms to form an adaptive PIR controller. Therefore, internal models of related periodic signals are incorporated into current and speed controllers. The PI term, the primary controller, creates the dc control and manages the dynamic performance of the system.

The resonant term’s output fundamental transfer function is

15 $$\:G\left(s\right)=\:\frac{{k}_{r}s}{{s}^{2\:}+{\omega\:}_{0}^{2}}.$$

From the above equation $\:{k}_{r}$ represents the coefficient of the resonant, and $\:{\omega\:}_{0}$ represents the resonant frequency. An additional phase fine-tuning term^30,45^ is added to (15), to further increase the resonant term’s stability.

16 $$\:G\left(s\right)=\:{{k}_{p}+k}_{r}\:\frac{s\text{cos}\phi\:-\:{\omega\:}_{0}\text{sin}\phi\:}{{s}^{2\:}+2{\omega\:}_{c}s+{\omega\:}_{0}^{2}}.$$

From the above Eq. (16) $\:{k}_{p}$ and $\:{k}_{r}$ represents the proportional and resonant gain, $\:{\omega\:}_{c}\:$indicates the cut-off frequency, $\:\phi\:\:$represents the compensation angle.

### PIR current controller design process

**Fig. 2:**
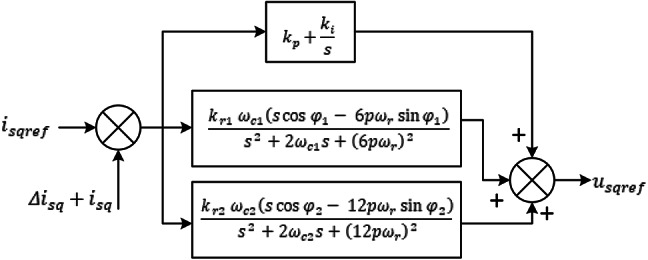
Current control loop design.

The design process for the existing PIR controller for $\:{i}_{sq}$ is displayed in Fig. 2. This idea can also be used to the PI controller. In order for $\:{i}_{sq\:}$to track *i*_*sqref*_ accurately and disregard disruptions, the controller must make sure of this. Two disturbances occur in the current loop when the *i*_*sd*_ *= 0* control approach is applied, as seen in Fig. 1 the dead time voltage *Δu*_*sq*_ and the back EMF $\:p\omega\:{\phi\:}_{dm}$.At the steady state, the frequencies of the ac components found in *i*_*sqref*_, *Δu*_*sq*_, and $\:\:p\omega\:{\phi\:}_{dm}$ are *6pω*_*r*_ according to the study in Section II. To enhance current controller performance, resonant terms with resonant frequencies of *nω*_*r*_ are thus included in tandem with the PI control term.

In order to balance complexity and performance, the maximum *n* in this work is fixed at 2; since higher order harmonics are not significantly affect speed harmonics. Furthermore, because subharmonics are associated with speed harmonics and disappear once speed harmonics are removed, it is not taken into account in this controller design. The q-axis PIR controller structure is depicted in Fig. 1, where the 6th and 12th order of the resonant cut-off frequency are denoted by *ω*_*c1*_ and *ω*_*c2*_, respectively; the 6th and 12th order of the resonant terms adjustment angles are denoted by $\:{\phi\:}_{1}$ and $\:{\phi\:}_{2}$, respectively; $\:{k}_{r1}$ and $\:{k}_{r2}$ is the resonant coefficients. The structure of the PIR controller for $\:{i}_{sd}$ is identical. Owing of the PMSM drive’s frequency-varying nature, the resonant controller should be able adapt to various operating conditions. To guarantee that the two resonant controllers can adjust their respective resonant frequency to correspond with the correct angular frequency, the rotation speed in electrical angle is thus used as the 1st order resonant frequency. Figure 2 displays the block diagram for a PMSM current control system with an adaptive PIR controller.

**Fig. 3:**
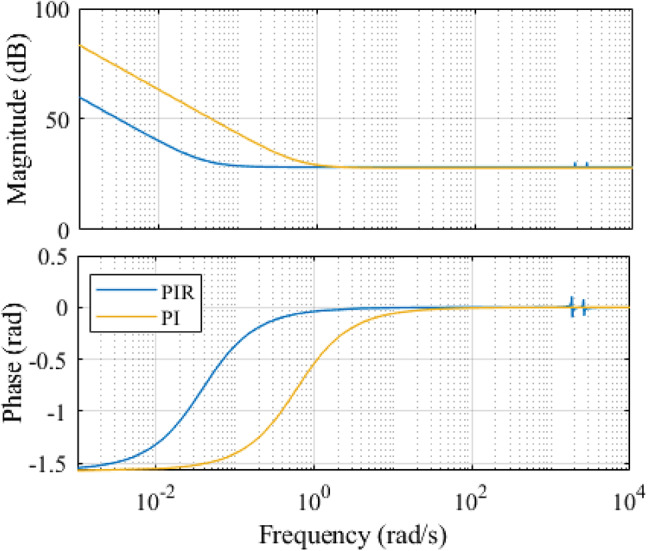
Analysis of current loop under PI and PIR control using bode plot.

A single operating point’s open loop bode diagram of conventional PI and an adaptive PIR controller is shown in Fig. 3. The control specifications for the simulations are as follows: The current loop’s bandwidth is 210 Hz; its *k*_*r1*_ and *k*_*r2*_ values are 200, 400, respectively; its resonant frequencies are $\:2\pi\:*60rad/sec$ and $\:2\pi\:*120rad/sec$, and its matching speed of the motor is 300r/min. It is demonstrated in Fig. 3 that every resonant phrase abruptly alters the systems phase and magnitude everywhere the resonant frequency. The resonant frequencies in Fig. 3 are $\:2\pi\:*60rad/sec$ and $\:2\pi\:*120rad/sec$, respectively, and all resonant term generates an unbounded open loop gain for magnitude. These incessant changes allow the current loop to correctly follow the references and totally reject disturbances of the same frequency. The tracking and anti-disturbance capabilities of the current loop can be enhanced since these two frequencies are the primary harmonic frequencies in the disturbances and reference signals. Additionally, it is evident that the resonant terms are highly selective, when the system frequency is far from the resonant frequency, their impacts are ignored.

### Determination of current controller parameters and analysis of stability

**Fig. 4:**
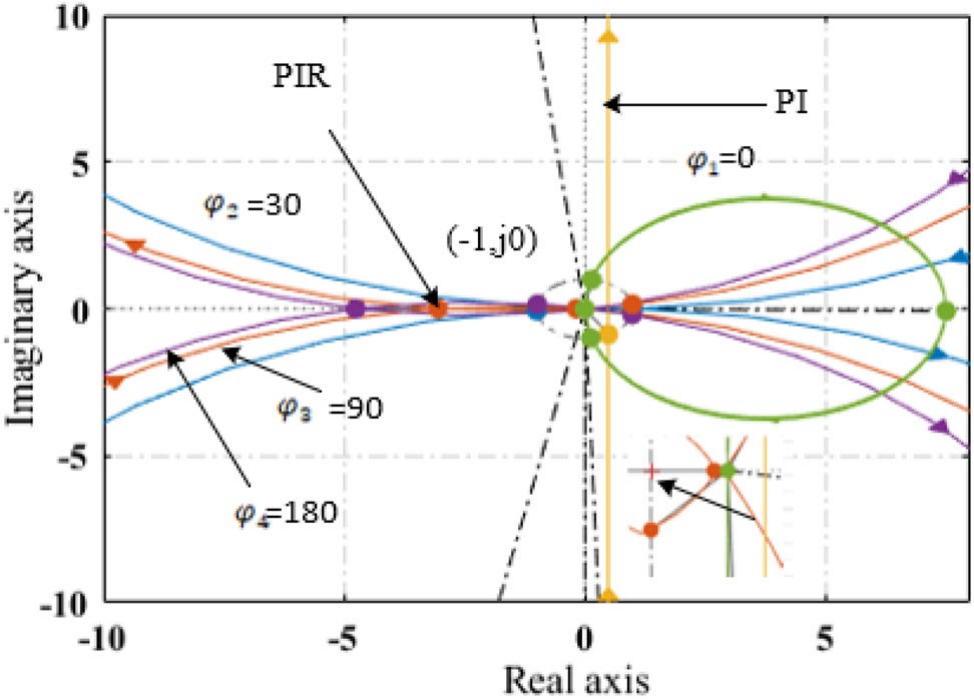
Current loop nyquist diagram with variable angle.

The resonant term is highly frequency selective, with its influence mostly concentrated on the resonant frequency, as shown in Fig. 2. On the other hand, *k*_*p*_ influences the resonant terms phase and amplitude at all frequencies. In order to stabilize the controller, the specifications of the current controller are adjusted in two stages: in first stage, the parameters of the PI terms are adjusted in the conventional manner, and then the parameters of each and every resonant term are identified. Since PI’s parameter design procedure has been examined in numerous studies, it won’t be addressed here. When is *f*_*c*_ equal to the internal current loop bandwidth, the tuning results appear including this: $\:{k}_{psd}=2\pi\:{f}_{c}{l}_{sd}$, $\:{k}_{psq}=2\pi\:{f}_{c}{l}_{sq}$ and $\:{k}_{isd}={k}_{isq}=2\pi\:{f}_{c}R$. The stability of the controller is analyzed in this paper using the nyquist diagram, and the current loop diagram is displayed in Fig. 4. For each resonant term, two parameters must be chosen: the resonant coefficient *k*_*rn*_ and the phase adjustment angle *φ*_*n*_. The former determines stability, while the latter impacts dynamic performance.

Figure 4 displays the current loop nyquist diagram of variable compensation angle $\:{\phi\:}_{cn}$ with constant resonant frequency to analysis the current loop stability of the system. The PIR controller’s stability might be explained as follows: if the PI controller is stable with the same PI settings, then the PIR controller’s stability is dependent on where the resonant arcs are in relation to the critical point (− 1, j0). Only when the critical point is not surrounded by arcs is the system stable. The distance between the critical point and the arcs indicates the system’s stable margin. A margin that is greater in distance is more stable.

The arc’s encirclement of the critical point is determined by the coordinates of the start and end points as well as *φ*_*n*_, the angle between the arc and the real axis when *ω* is getting close to the resonant frequency, as illustrated in Fig. 4.

*ϕ*_*n*_ is calculated as follows

17 $$\:{\theta\:}_{n}=\:\frac{\pi\:}{2}+\:{\phi\:}_{n}-arctan\frac{{L}_{sq6n{\omega\:}_{r}}}{R}-arctan{T}_{1}6np{\omega\:}_{r}.$$

Form the above equation *T*_*1*_ is the overall delay on inverter and controller. It is evident that *T*_*1*_ influences the system’s stability, which can be enhanced by modifying$\:\:{\phi\:}_{n}$.

18 $$\:{\phi\:}_{n}=\:\frac{{\phi\:}_{n}\text{max}-\:{\phi\:}_{n}min}{2}\:$$.

When the motor speed is increased it could be increased the value of $\:{\phi\:}_{n}\text{max}\:\text{a}\text{n}\text{d}\:{\phi\:}_{n}min$. In addition to that their values depend on the resistance and inductance values.

### Parameter tuning method of resonant controller

The control rule is supposed to be the only factor influencing the current’s transient behavior in the scheme under study. The only purpose of the PIR is to offset the ac disruption. The PIR parameter selection must meet the next two requirements in order to meet the aforementioned conditions. First, the PIR only exhibits a significant gain at the chosen resonant frequency. The second requirement is that the gain be sufficiently high to totally suppress the ac disturbance.

**Fig. 5:**
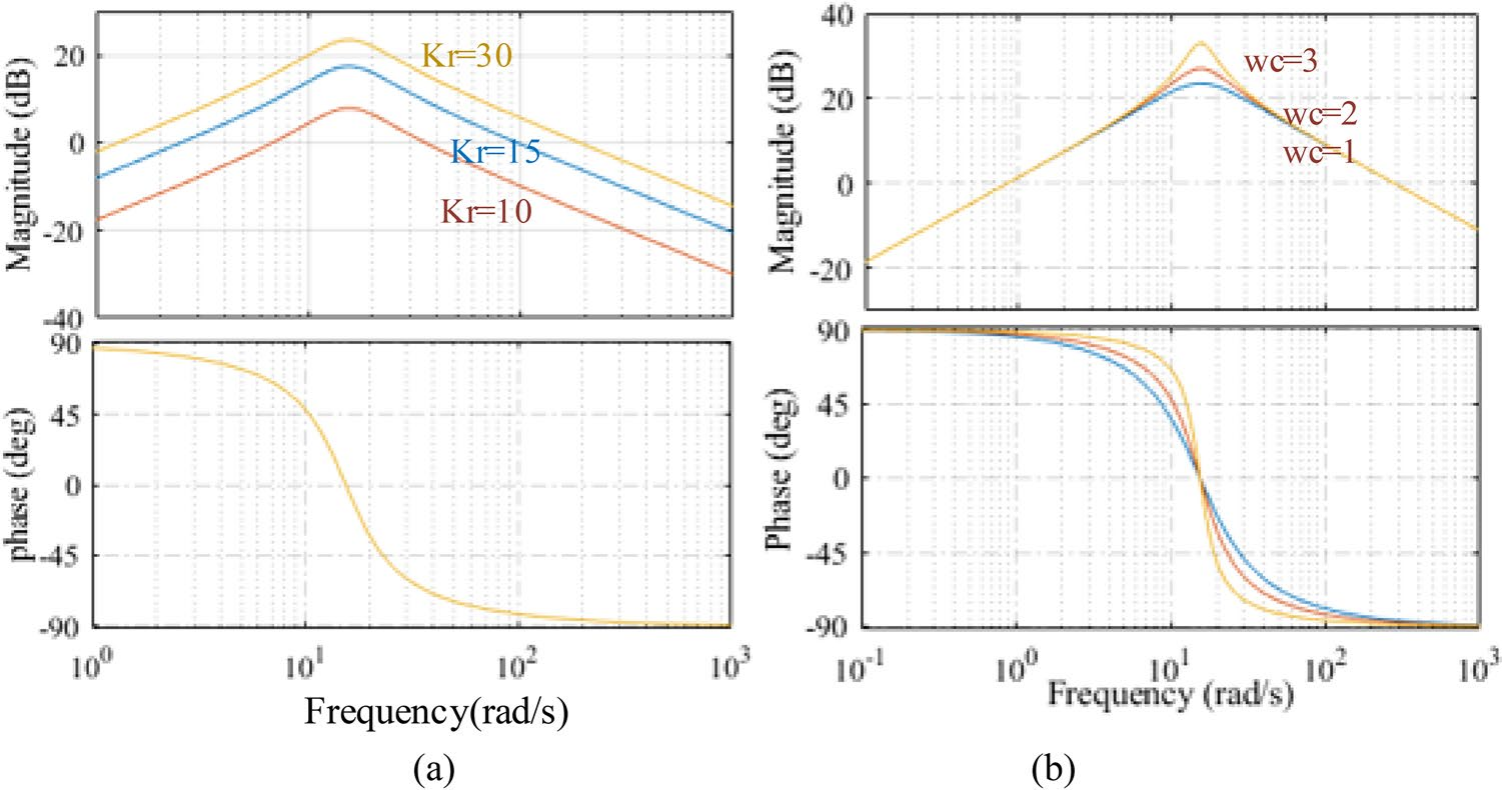
Bode characteristics of resonant controller parameter while (**a**) keeping *k*_*r*_ variable and $\:{\omega\:}_{c}\:$constant (**b**) keeping *k*_*r*_ variable and $\:{\omega\:}_{c}$

Three parameters must be chosen for each resonant term: the resonant coefficient *(*$\:{k}_{r}$*)* and the phase adjustment angle *(*$\:{\phi\:}_{cn}$*)*and cut-off frequency $\:{\omega\:}_{c}$. The bode plot of Fig. 5, which shows the current loop diagram, is used to analyse the controller’s stability with $\:={45}^{0}\:$. The simulation’s parameters are the same as those in Fig. 2. Figure 5 shows its features with varying *k*_*r*_ and $\:{\omega\:}_{c}$. As Fig. 5(a) illustrates, it is evident that the open-loop gain and bandwidth at the resonant frequency rises with *k*_*r*_. The PMSM parameter mismatch has an impact on PIR performance since *k* is set to *R*/*L*(R is the resistance and L is the inductance) in PMSM drivers. By raising $\:{\omega\:}_{c}$, from $\:{\omega\:}_{c}$= 1,2,3 hence the controller gain is increased with increase of $\:{\omega\:}_{c}$, as illustrated in Fig. 5b. Ideally, control performance improves with increasing *k*_*r*_ and $\:{\omega\:}_{c}$ [31]. On the other hand, an excessively high *k*_*r*_ may cause instability in the system performance, and an excessively large or small $\:{\omega\:}_{c}$ may impact the frequency-selecting feature. Therefore, *k*_*r*_ and $\:{\omega\:}_{c}\:$kept has careful consideration.

The PIR tuning approach can be summed up as follows based on the two characteristics: In order to mitigate the impact of PIR on signals at frequencies other than the chosen frequency, we first choose an appropriate *k*_*r*_. Then, to successfully suppress the ac disturbance, a small $\:{\omega\:}_{c}$ should be used. A system that is overly sensitive to the input signal’s frequency due to a small$\:{\:\omega\:}_{c}$, on the other hand, it may unstable. Thus, the system stability and antidisturbance performance should be balanced while choosing$\:\:{\omega\:}_{c}$. The parameter mismatch may also cause *k*_*r*_ to be less than the value determined using PMSM parameters, which would decrease the controller bandwidth at the chosen resonant frequency and diminish its capacity to resist ac disturbances. Hence, to overcome the stability issues and impact on the frequency varying feature *k*_*p*_ *= 3.01*,* k*_*r*_ = 25 and $\:{\omega\:}_{c}$ = 1.3dB is selected for the proposed adaptive PIR control system for steady state and dynamic performance. The resonant controller should be able to adjust to various operating situations because of the PMSM drive’s frequency-varying nature.

### PIR speed controller design process

It is comparable to equivalent interruption of the outer speed loop in terms of $\:{i}_{sqref}\:$and$\:\:{i}_{sq}$. Moreover, there are two interruptions in the outer speed loop if the inner current loop is considered first-order inertia. As depicted in Fig. 1, they are the cogging torque $\:{T}_{cog}\:$ and the current measurement errors$\:{\:\varDelta\:i}_{q}$. Since load torque is uncontrollable, it is not considered here. In accordance to the investigation in Section II, the frequencies in $\:{T}_{cog}$ are *nN*_*co*_*ω*_*r*_, and those in $\:{\varDelta\:i}_{sq}\:$ are *ω*_*r*_ and *2ω*_*r*_. Using resonant terms with these frequencies, the speed PIR controller performs better in terms of rejection of disturbances. The determined flux harmonics are a considerable contributor to speed harmonics. After rejection of the harmonics, can get a steady state performance, hence it can be written as,

19 $$\:{i}_{sq}\left(t\right)=\:\frac{{T}_{L}+B{\omega\:}_{e}}{1.5p\:\left[{\phi\:}_{sd0}+\sum\:_{n=1}^{\infty\:}\text{cos}\left(np(t\right)\right]}.$$

**Fig. 6:**
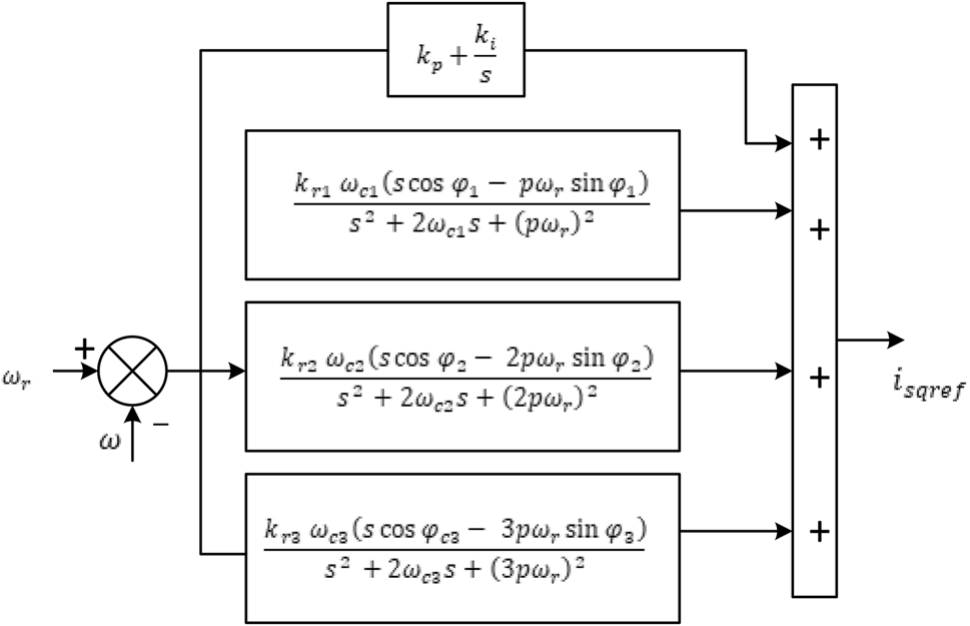
PIR speed controller design.

It suggests that components with frequency of *nω*_*r*_ must be present in $\:{i}_{sq}\left(t\right)$ to maintain a constant speed. This indicates that the speed PIR controller needs the resonant terms with those resonant frequencies. In low and high speed PMSM drives EV, lower frequency distortions are a primary source of declining performance. Due to rotor inertia and speed measurement, high frequency speed harmonics are often insignificant. Consequently, this research just examines the cogging torque and the fundamental frequency of harmonics present in flux. Additionally, the cogging torque period and the flux harmonic period match, based on the number of slot and poles of the motor. Figure 6 represents the outer speed loop PIR controller it has three resonant terms where $\:{k}_{r1,\:}\:{k}_{r2,\:}$ and $\:{k}_{r3}$ are the resonant output terms. Flux harmonics and the cogging torque period occur at the same time.

### Speed PIR controller stability analysis and parameter tuning

The outer speed loop PI controller specifications are determined in the similar steps as the existing controller. The PI terms parameters are initially established using conventional methods. Finding the parameters of each resonant term then ensures controller stability and good dynamic.

**Fig. 7:**
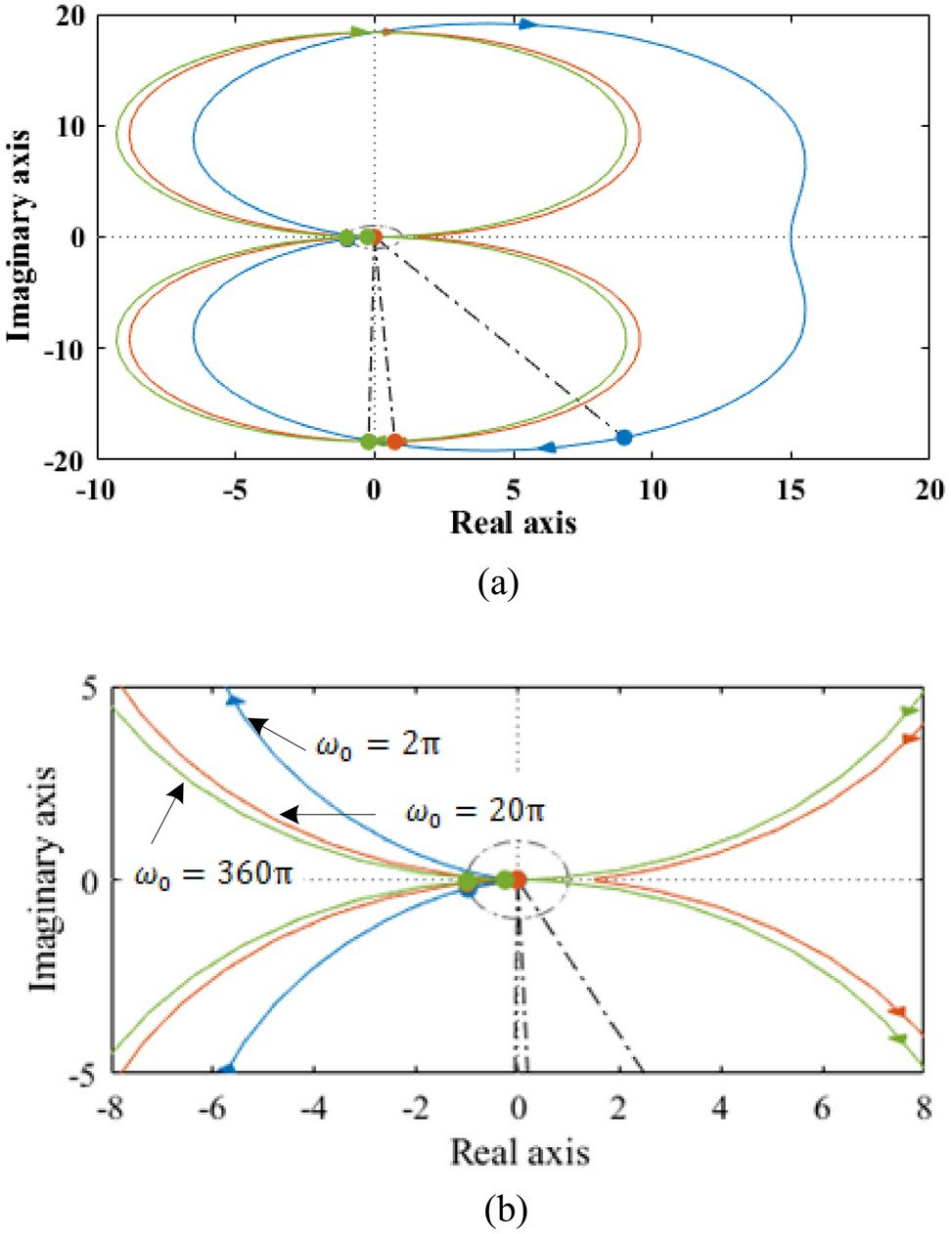
Speed loop nyquist plot with various resonant frequency (**a**) original view (**b**) zoomed view.

The speed loop’s nyquist diagram with various resonance frequencies is displayed in Fig. 7a and b. It shows the phase adjustment angles as 90◦, 0◦, and 120◦, respectively. For simplicity, only the 1st order resonant term is displayed here. This is because each resonant term primarily acts around its resonance frequency, this figure shows how the relative locations of the beginning and final points to the important point, in addition to alter dramatically as the resonant frequency rises.

### Conditional integral term

The speed reference $\:{\omega\:}_{r\:}$is used to construct the frequencies of resonant term in outer loop speed control and inner current loop an adaptive PIR controllers. The resonant terms can effectively suppress the disturbances because in a stable condition, the frequencies of the resonant term overlap with them. Nevertheless, the resonant terms should lose this capability in dynamic when there is a significant discrepancy between motor speed and its respective reference. Resonant term output would increase dramatically and reduce the control influence since the controller input signals contain a lot of ac components in motor dynamics. Therefore, in this case, a conditional integral technique is applied. When the absolute difference *ω* and $\:{\omega\:}_{r}$exceeds a threshold, the resonant terms in speed loop and current loop PIR controllers are turned off. Their values are instantaneously kept as zero. These resonance terms are engaged again after the dynamics. When determining when the resonant terms are triggered again, the dynamics caused by a step change in the load and reference speed are analyzed. To simplify the analysis, the delay of the speed feedback channel is ignored, and the current loops are considered ideal. One way to represent the closed-loop transfer function of the speed loop is as follows,

20 $$\:{G}_{io}\left(s\right)=\:\frac{1.5p{\phi\:}_{dm}({k}_{ps}+\:{k}_{is})}{{J}_{s}^{2}+\left(1.5p{\phi\:}_{dm}{k}_{ps}+B\right)s+1.5p{\phi\:}_{dm}{k}_{is}}.$$

Substituting by using the PI tuning instructions in Section III-D (20) yields

21 $$\:{G}_{io}\left(s\right)=\frac{{\omega\:}_{n}s+{\omega\:}_{n}^{2}}{{s}^{2}+\left({\omega\:}_{n}+\frac{B}{J}\right)s+{\omega\:}_{n}^{2}}.$$

When friction is ignored, the reaction of (21) to a step change in *ω*_*r*_ can be written as

22 $$\:{H}_{I}\left(t\right)=\:\omega\:-\:{\varDelta\:\omega\:}_{r\:}\frac{2}{\sqrt{3}}{e}^{-\frac{1}{2}{\omega\:}_{n}t}sin\left(\frac{\sqrt{3}}{2}{\omega\:}_{n}t+\frac{\pi\:}{6}\right).$$

From the above equation where $\:{\varDelta\:\omega\:}_{r\:}$is change in reference speed. By following similar steps, the response step to $\:{\varDelta\:T}_{L\:}$represents the change in load torque is found and it represented as (23)

23 $$\:{H}_{2}\left(t\right)=\:\omega\:-\:\frac{{\varDelta\:T}_{L}\:}{J{\omega\:}_{n}}\frac{2}{\sqrt{3}}{e}^{-\frac{1}{2}{\omega\:}_{n}t}sin\left(\frac{\sqrt{3}}{2}{\omega\:}_{n}t\right).$$

The adjusting time of$\:{\:H}_{I}\left(t\right)$,$\:\:{t}_{s1\:}$ is defined as the smallest amount of time between the start of the dynamics and the point at which error speed decays into the region of ± 1.9%$\:{\varDelta\:\omega\:}_{r}$, and the adjusting time of $\:{H}_{2}\left(t\right),{t}_{s2\:}$ is defined as the smallest amount of time between the start of the dynamics and the point at which speed error decays into the region of ± 1.5%$\:{\varDelta\:T}_{L}$/$\:\:J{\omega\:}_{n}$. The envelope line of (22) and (23), respectively, can be used to evaluate the values of $\:{t}_{s1\:}$ and$\:{\:t}_{s2\:}$, i.e.,

24 $$\:{t}_{s1\:}={\:t}_{s2\:}\approx\:\:\frac{7.9}{{\omega\:}_{n}}.$$

According to Eq. (19) the motor dynamics are essentially over 7.9/$\:{\omega\:}_{n}$ sec. However, the power restriction of a realistic inverter was not considered in (20) or (21). When speed exceeds 0.9$\:{\omega\:}_{r}$, the resonant terms are activated 7.9/$\:{\omega\:}_{n}$ seconds after the initial occurrence. This way, the resonant and disturbance frequencies are consistent and the side effect is avoided.

### Implementation of digital resonant controller

There is a continuous transfer function (15). Discretization is required in order to apply the proportional resonant current controller to the EV PMSM control system.

The bilinear transformation is follows

25 $$\:s=\:\frac{2\left(1-{z}^{-1}\right)}{{T}_{S}\left(1-{z}^{-1}\right)}.$$

Substitute (25) into (15), yields

26 $$\:G\left(z\right)=\:\frac{{b}_{0\:}+{b}_{1}{z}^{-1}+{b}_{2}{z}^{-2}}{1+{a}_{1}{z}^{-1}+{z}^{-2}}.$$

Where

$$\:{\:\:\:b}_{0}=\:{k}_{r}\frac{2{P}_{s\:}cos\phi\:-\:{P}_{s}^{2}{\omega\:}_{0}sin\phi\:}{4+\:{\omega\:}_{0}^{2}{P}_{s}^{2}}$$

$$\:{b}_{1}=\:-{k}_{r}\frac{2\:{P}_{s}^{2}{\omega\:}_{0}sin\phi\:}{4+\:{\omega\:}_{0}^{2}{P}_{s}^{2}}$$

$$\:{b}_{2}=\:-{k}_{r}\frac{2{P}_{s\:}cos\phi\:+{P}_{s}^{2}{\omega\:}_{0}sin\phi\:}{4+\:{\omega\:}_{0}^{2}{P}_{s}^{2}}$$

$$\:{a}_{1}=\:\frac{2\:{P}_{s}^{2}{\omega\:}_{0}^{2}-8}{4+\:{\omega\:}_{0}^{2}{P}_{s}^{2}}.$$

From the above equation $\:{P}_{s\:}$ is the sampling period. By using reference speed resonant coefficient can be directly calculated.

The equation of the controller is obtained,

27 $$\:y\left(t\right)=\:{k}_{p}e\left(t\right)+{b}_{0}e\left(t-1\right)+{b}_{1}e\left(t-2\right)+{b}_{2}e\left(t-4\right)-{a}_{1}e(t-5).$$

### Causes and effect of the proposed system

This section describes the causes and effect of the proposed system. It includes what is causes and effect of inductance variation and effect of 3-dBcut off frequency variation.

#### Effect of inductance variation causes increased harmonics

Magnetic saturation causes the inductance term $\:\varDelta\:L$ to fluctuate while the machine is operating. $\:\varDelta\:L$‘s real value is expressed as follows if it is anticipated to drop by a percentage of γ

28 $$\:\varDelta\:L=\frac{{\omega\:}_{r}}{\omega\:}(1-\gamma\:)img\varDelta\:L.$$

Likewise torque ripple and system vibration caused by small changes in inductance variation is given below,

29 $$\:{t}_{h}=\:\frac{{\left(\varDelta\:L{\:i}_{sd}\right)}^{2}+\varDelta\:L{\:i}_{sd}+{\left(\varDelta\:L{\:i}_{sq}\right)}^{2}}{{\left(\varDelta\:L{\:i}_{sd}\right)}^{2}+{\left(\varDelta\:L{\:i}_{sq}\right)}^{2}}\gamma\:\:X\:\sum\:_{n}\left[{E}_{n}\text{cos}\left(n\theta\:\right)+{F}_{n\:}\text{s}\text{i}\text{n}\left(n\theta\:\right)\right].$$

From the above equation $\:\varDelta\:L$ is the change in inductance, *E* and *F* is the fourier coefficients. The inductance variation causes vibration in the drive system. To mitigate the vibration and torque ripples due to inductance variation it should be careful consideration on the values of the inductance.

#### Effect on 3-dB cut-off frequency causes instability

A degree of flexibility in the design of the resonant frequency responses seems to be possible with the low-pass 3-dB cut-off frequency. As a common indicator of a filter’s bandwidth, expanding its size would seem to increase the impact of the high gain produced by the resonant term. As a result, the system would seem to be less susceptible to changes in the fundamental frequency, increasing the resilience of the regulator. Unfortunately, this benefit is not present in practice, as demonstrated by setting the gain. It should eliminate the gain variation in (15) and makes it easier to see the impact of changes. The frequency analysis is made using (15). The gain at the resonant frequency of the damped resonant term produced by the transformation is provided by$\:\:s=j\omega\:$

30 $$\:G\left(j\omega\:,\:{\omega\:}_{c}\right)=\:{{k}_{p}+k}_{r}\:\frac{j\omega\:\text{cos}\phi\:-\:{\omega\:}_{0}\text{sin}\phi\:}{{\left(j\omega\:\right)}^{2\:}+2{\omega\:}_{c}j\omega\:+{\omega\:}_{0}^{2}}.$$

Simplified equation of the above (30) is yield,

31 $$\:G\left({j\omega\:}_{0},\:{\omega\:}_{c}\right)=\:{{k}_{p}+k}_{r}\:\frac{{\omega\:}_{0}(j\text{cos}\phi\:-\text{sin}\phi\:)}{2j{\omega\:}_{c}{\omega\:}_{0}}.$$

To calculate $\:{\omega\:}_{0}$in both side the equation yield

32 $$\:G\left({j\omega\:}_{0},\:{\omega\:}_{c}\right)=\:\frac{{{k}_{p}+k}_{r}}{2{\omega\:}_{c}}\:\frac{j\text{cos}\phi\:-\text{sin}\phi\:}{j}.$$

After simplification the equation yield

33 $$\:G\left({j\omega\:}_{0},\:{\omega\:}_{c}\right)=\:\frac{{{k}_{p}+k}_{r}}{2{\omega\:}_{c}}(\text{cos}\phi\:-\text{sin}\phi\:).$$

The magnitude of (30) can expressed as follows

34 $$\:\left|G\left({j\omega\:}_{0},\:{\omega\:}_{c}\right)\right|=\:\frac{{{k}_{p}+k}_{r}}{2{\omega\:}_{c}}\sqrt{({\text{cos}}^{2}\phi\:-{\text{sin}}^{2}\phi\:)}=\:\frac{{{k}_{p}+k}_{r}}{2{\omega\:}_{c}}.$$

Thus, it is evident that changing the peak amplitude without changing the resonant frequency of the resonant terms the gain in resonant frequency is affected. Due to the variation in the cut-off frequency of the resonant controller the system becomes more sensitive to disturbance, hence it causes instability, steady state error, and decrease the efficiency of the PMSM based EV drive. Hence, the value of cut-off frequency should contain careful consideration.

## Results and verifications

**Fig. 8:**
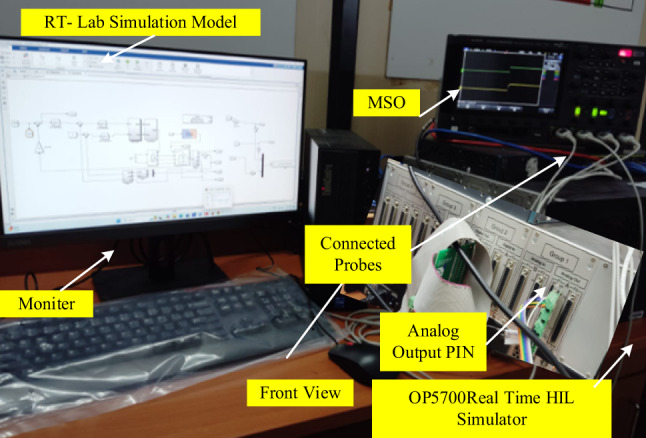
Real time simulator setup.

To analysis the overall performance of PMSM drive systems simulation and experiments (HIL) are taken out with the specification listed in Table 1. Figure 8 shows the complete layout of the HIL setup of the control scheme using OPAL-RT5700 platform.

**Table 1:** Specifications of the respected system^47^.

Parameters and unit	Notations	Values
DC link voltage (v)	$\:{v}_{dc}$	311
Rated power (w)	$\:{P}_{r}$	6400
Rated speed (r/min)	$\:{N}_{r}$	4000
Rated torque (Nm)	$\:{T}_{e}$	30
Flux linkage (Wb)	$\:{\psi\:}_{f\:\:}$	0.035
*d* - axis inductance (mH)	$\:{L}_{d}$	16
*q* - axis inductance (mH)	$\:{L}_{q}$	16
Resistance in Stator (Ω)	$\:{R}_{s}$	0.0852
Number of Pole pair	*p*	4
Sampling period (µs)	$\:{P}_{s}$	11
Inertia(kgm^2^)	*J*	3.74

HIL systems are frequently used in engineering systems for pre-prototyping experiments using real-time simulation. Prototypes can be swiftly created and synchronised using stacks. To operate the system at the precise clock time, the machine and controller are mounted in OPAL-RT. This system is dynamic in real-time because to the high OPAL-RT sample speed, which ranges from nano to microsecond. The RT-LAB’s digital simulator commands are managed by the user PC. RT-LAB is used for editing, building, loading, and running the prototype. Table 2 covers the specifications for the HIL stack and the ability to execute real-time systems. The specification of the HIL setup is depicted in Table 2.

**Table 2:** HIL specification^48^.

Device name	OP5700
Field Programmable Gate Arrays(GPGA)	Xlinx^®^ vertex^®^ FPGA on VC707 board processing speed: 200ns-2µs
Input and output lines	256 lines, routed to eight analog or digital, 16 or 32 channels
High speed communication ports	Up to 5 GBps
Input and output Connectors	Four panels of BD37 interfaces
PC interfaces	Four panels of RJ45 interfaces
Monitoring interfaces	Standard PC connectors
Power rating	Input:100-240VAC,50-60HZ,10/5A Power:600 W

In this proposed system drive, first create the model and run the system on the MATLAB/Simulink platform. Next, added a few OPAL-RT libraries based on the OPAL-RT simulator’s OP5700 configuration. After that, the modified model is transferred to the real-time simulator-OP5700 kit (for data recording and I/O management) via Ethernet (the link between the host PC and simulator). Lastly, the model’s RT output is extracted via the analog/digital interface from the digital storage oscilloscope.

### Simulation and experimental verifications

The system is evaluated using MATLAB/Simulink to assess the effectiveness of the proposed control technique, and the results are compared with experimental findings. For comparison, conventional PI^34^ controllers are also implemented under the same specifications. The effectiveness of the proposed control method is analyzed against the conventional PI controller in accordance with the various system parameters it includes speed, torque, and current. By eliminating the need for costly computations such coordinate transformations, the proposed PIR controller speeds up the vehicle controller’s computation and improves its resilience. Similarly, the outer PIR control loop lowers chattering effects, improves anti-disturbance performance, and accelerates the EV’s dynamic reaction to assure superior speed and torque management.

#### Test and verification of different speed and load torque scenario

To evaluate the outer speed loop performance of the proposed controller this section includes the following categories (i) During low speed with maximum load torque (ii) During high speed with absence of load (iii) different acceleration and deceleration of speed. Those categories detailed explanation is given in below section.

##### Mode 1: during low speed with maximum load torque

**Fig. 9:**
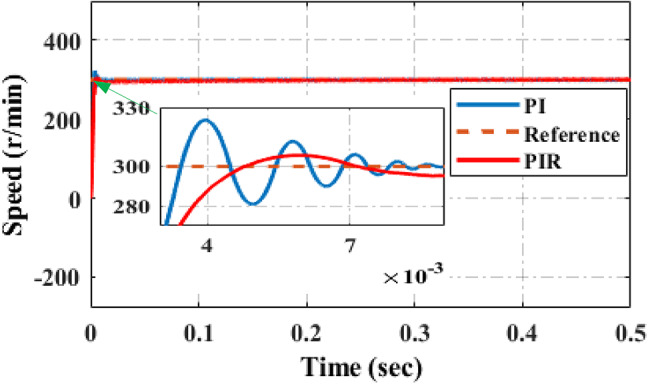
Speed performance under low speed with maximum load.

The simulation results of PIR and PI control systems are displayed in Fig. 9 to confirm the speed loop performance of proposed controller. In this simulation results the speed of 300r/min is applied to the motor at 15Nm load torque condition. It is evident that the output of speed controller under PI control has more distortion, undershoot and overshoot. The harmonics are especially noticeable when the motor is operating at a low speed or with a lot of loads. They are significantly suppressed by PIR control. It can reduce undershoot and overshoot of the speed waveform when the controller operates under an adaptive PIR control technique.

Experimental verification of an adaptive PIR control mechanism is conducted for the similar use cases that are examined in simulation verification. To perform experimental verification, a hardware-in-loop (HIL) simulator, the OPAL-RT5700 platform, is used. Figure 8 shows the RT-lab HIL configuration for the system under consideration. In order to verify the performance of the proposed PIR controller, the conventional PI controllers are also tested into the OPAL-RT5700 simulator in order to verify the system’s performance outcome.

**Fig. 10:**
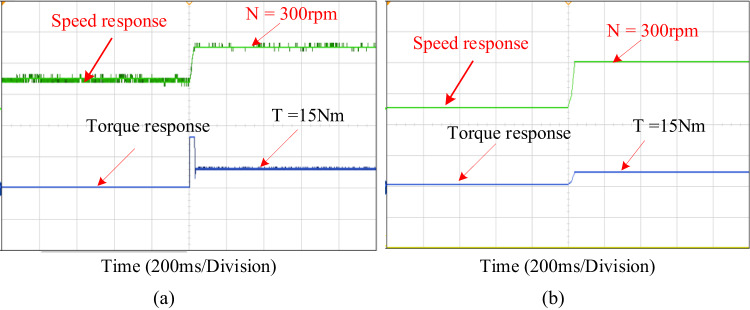
Experimental speed performance under low speed with maximum load.

The experimental results of PI and proposed PIR control systems are depicted in Fig. 10 to confirm the speed loop performance of proposed controller. In these experimental results the speed of 300r/min is applied to the motor at 15Nm load torque condition. It is evident that the speed characteristics under PI control has more disturbances and the system rise time is high as shown in Fig. 10a. The disturbances are especially noticeable during the machine is operating at a lower speed or with a lot of loads. They are significantly suppressed by an adaptive PIR control as illustrated in Fig. 10b. It can conclude that an adaptive PIR controller can considerably reduce the speed fluctuation, rise time, torque ripple, overshoot and undershoot in both simulation and experimentally. Hence the proposed system performance and efficiency is increased.

##### Mode 2:during high speed without load

**Fig. 11:**
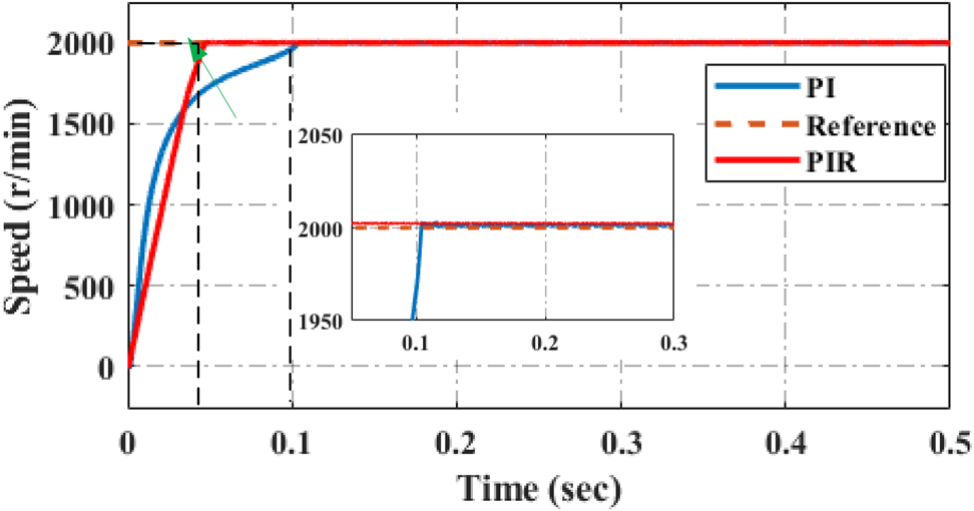
Speed performance under high speed and without load.

In this simulation results the speed of 2000r/min is applied to the machine without load condition as illustrated in Fig. 11. It is evident that the speed characteristics waveform under PI control has more fluctuation and takes more time to reach the reference speed due to its absence of resonant gain and its fixed frequency. Due to its resonant gain at fundamental frequency compared to PI controller proposed controller acceleration speed is high and it takes less time to reach reference speed The fluctuation and rise time are especially mitigated during the motor is operating at a high speed or with no load torque. It is significantly suppressed by PIR control. The acceleration speed of the proposed system high hence it takes less rise time to reach the reference speed. The overall performance analysis under high speed with absence of load is illustrated in Table 3. From this table compared to PI controller an adaptive PIR controller gives best result it includes rise time, settling time, overshoot and undershoot.

**Table 3:** Overall system performances under high speed with no load.

Control techniques	Rise time (sec)	Settling time (sec)	Overshoot (%)	Undershoot (%)
PI control^46^	0.0045	0.05	3.0	25
PIR control	0.0031	0.009	1.5	1.4

**Fig. 12:**
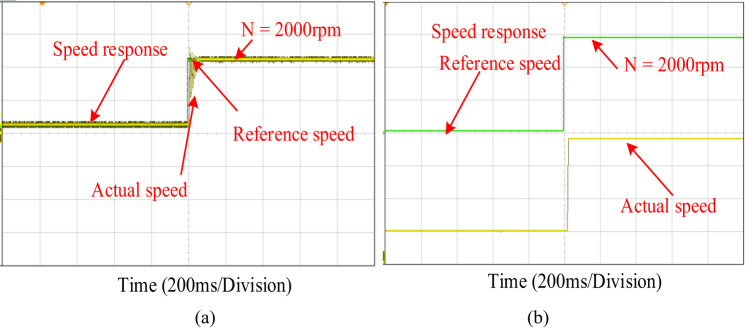
Experimental performance verification of high speed and without load (**a**) PI control (**b**) PIR control.

In this experimental result half of the rated speed of 2000r/min is applied to the motor without load torque condition as shown in Fig. 12. It is evident that the speed characteristics waveform during PI control has more steady state error, settling time, rise time and overshoot, undershoot oscillation as shown in Fig. 12a. The fluctuation, settling time steady state error especially mitigated when the motor is operating with a proposed control system as depicted in Fig. 12b. It can be summarized that the proposed controller can gives best performance outcomes in both simulation and experimentally.

##### Mode 3: different acceleration and deceleration of speed condition

**Fig. 13:**
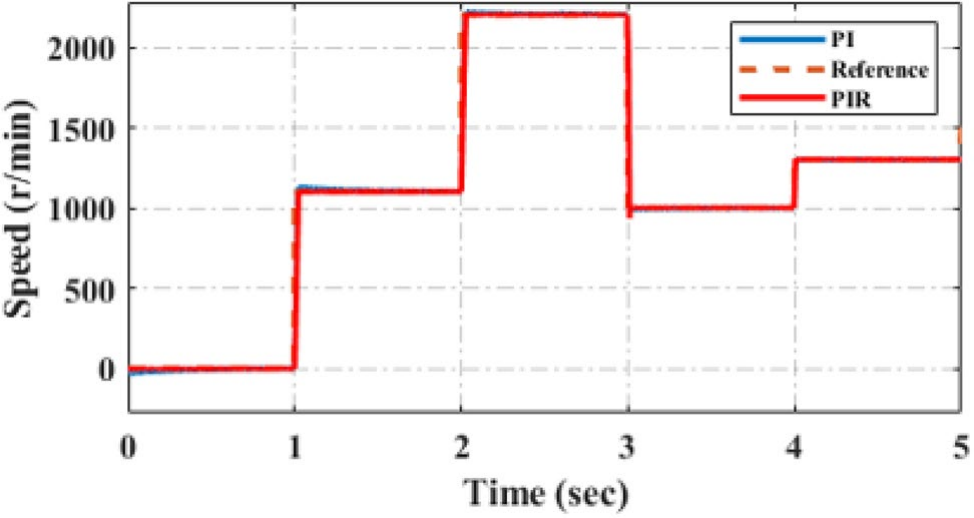
Analysis of dynamic response of speed.

**Fig. 14:**
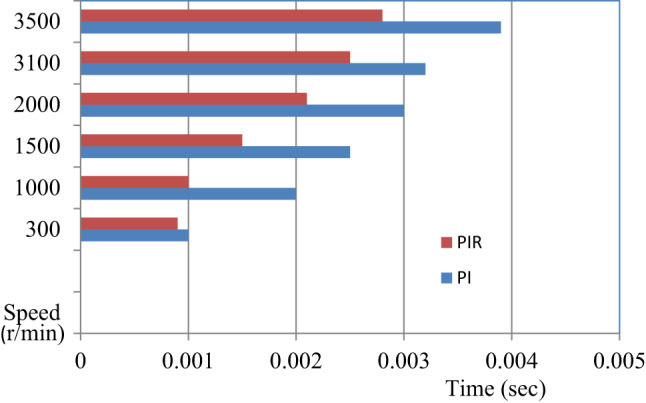
Graphical representation of performance analysis under speed rise time.

Figure 13 displays the speed response of EVs during acceleration and deceleration conditions. The motor is started at a 0r/min at 15Nm torque up to 1 s time interval. At t = 1 s the speed is increased from 0r/min-1200r/min up to t = 2 s the speed remains constant. At t = 2 s the speed is increased from 1200r/min-2300r/min until t = 3 s the motor runs in same speed. At t = 3 s the motor goes to deceleration condition the motor speed is decreased from 2300r/min- 1000r/min until the time reaches t = 4 s. at t = 4 s again the motor speed is increased from 1000r/min – 1250 r/min. throughout this acceleration and deceleration of the motor load torque remains constant. Figure 13 illustrates, compared to conventional PI controller, how the proposed approach may swiftly follow the change in acceleration instruction with the least amount of steady-state error, so offering drivers who must accelerate, decelerate and pass a safety buffer. The overall performance rise time of the speed is tabulated in Table 4 and the respective graphical representation are illustrated in Fig. 14.

**Table 4:** Quantitative analysis of speed rise time performance comparison.

Speed	Rise time (s)	
	PI control^46^	PIR control
300	0.001	0.0009
1000	0.002	0.001
1500	0.0025	0.0015
2000	0.003	0.0021
3100	0.0032	0.0025
3500	0.0039	0.0028

**Fig. 15:**
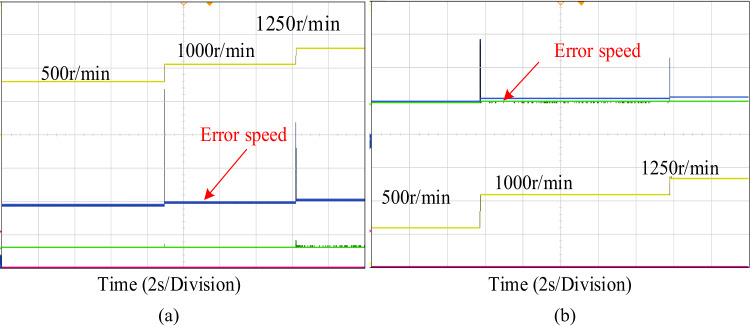
Dynamic speed variation under (**a**) PI control (**b**) PIR control.

Figure 15 displays the speed characteristics response of machine’s during acceleration and deceleration circumstances. The machine starts at 500r/min at 15Nm torque up to sometime interval. After the speed is increased from 500r/min-1000r/min the speed remains constant. The speed is increased from 1000r/min-1250r/min the machine runs in same speed, throughout this dynamic speed verification of the machine load torque remains constant. Figure 15a illustrates, compared to conventional PI controller, how the proposed PIR control system as demonstrated in Fig. 15b may swiftly follow the change in acceleration instruction with the least amount of steady state error, so offering drivers who must accelerate, decelerate and pass a safety buffer. Hence it is evident that the proposed controller gives best results in both simulation and experimental verification.

#### Test and verification of current loop performance

**Fig. 16:**
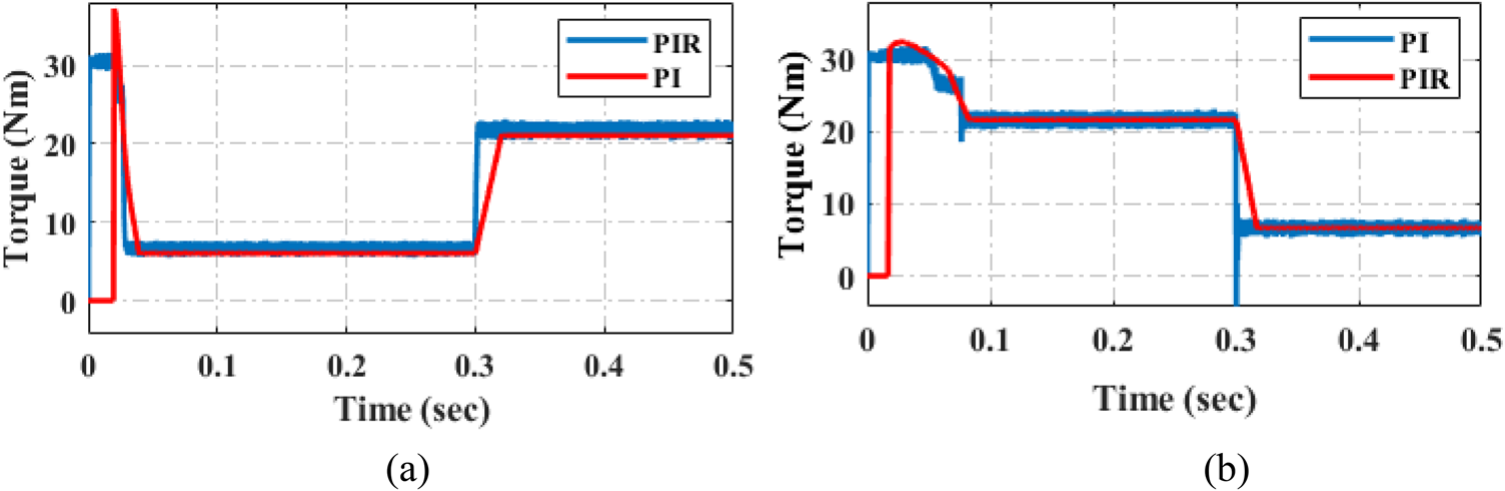
Performance analysis of load torque under (**a**) load torque increased (**b**) load torque decreased.

This section assesses the current and load torque performance of the PI and proposed PIR control scheme are illustrated in Fig. 16. In the first test case the machine starts up at 300 r/min when it is loaded at 5 Nm. The machine raises the load to 20 Nm in 0.3 s is depicted in Fig. 16a. During torque verification, the EV machine speed remains unchanged. In the second test case the machine starts up at 300 r/min when it is loaded at 20 Nm a. The machine decreases the load to 5 Nm in 0.3 is depicted in Fig. 16b. During torque verification, the EV machine speed remains unchanged. From this result compared to PI controller the proposed controller has decreased torque harmonics and after the load varied condition it reaches the steady state condition earlier.

**Fig. 17:**
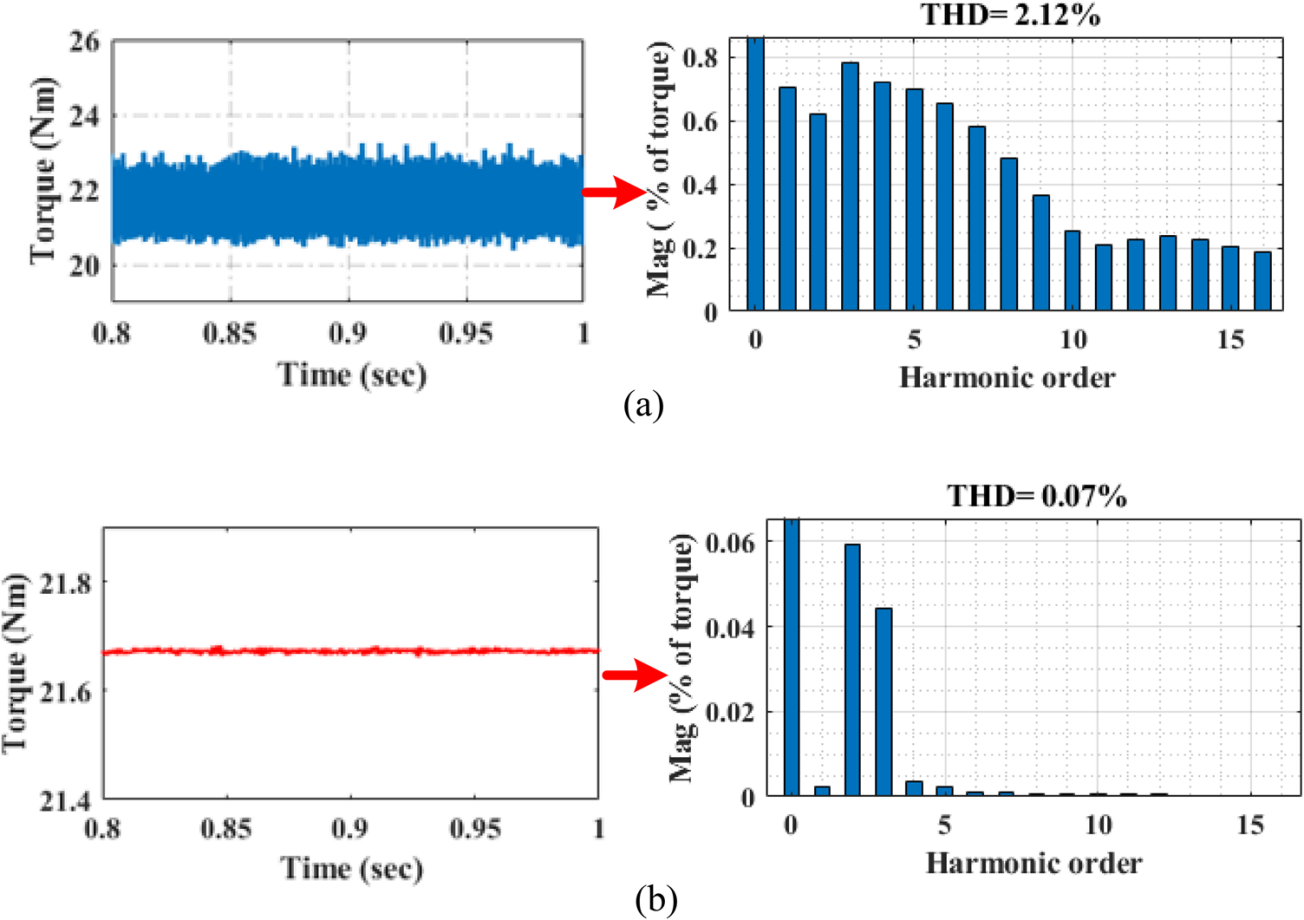
Performance comparison of torque under (**a**) PI control method (**b**) proposed control method.

Figure 17 displays the torque performance analysis of PI and the proposed adaptive PIR controller under 20Nm torque at 2000r/min. According to Fig. 17a, which shows torque performance while the system is operating under PI control techniques, the torque harmonics THD value is 2.12%. The torque harmonic value of the proposed adaptive PIR controller is THD 0.07%, as shown in Fig. 17b. These findings suggest that the proposed controller outperforms the conventional PI controller in terms of THD values. The performance analysis indicates that the proposed system has reduced noise and vibration, reduced the value of THD, and increased overall performance and efficiency when compared to the PI controller.

**Fig. 18:**
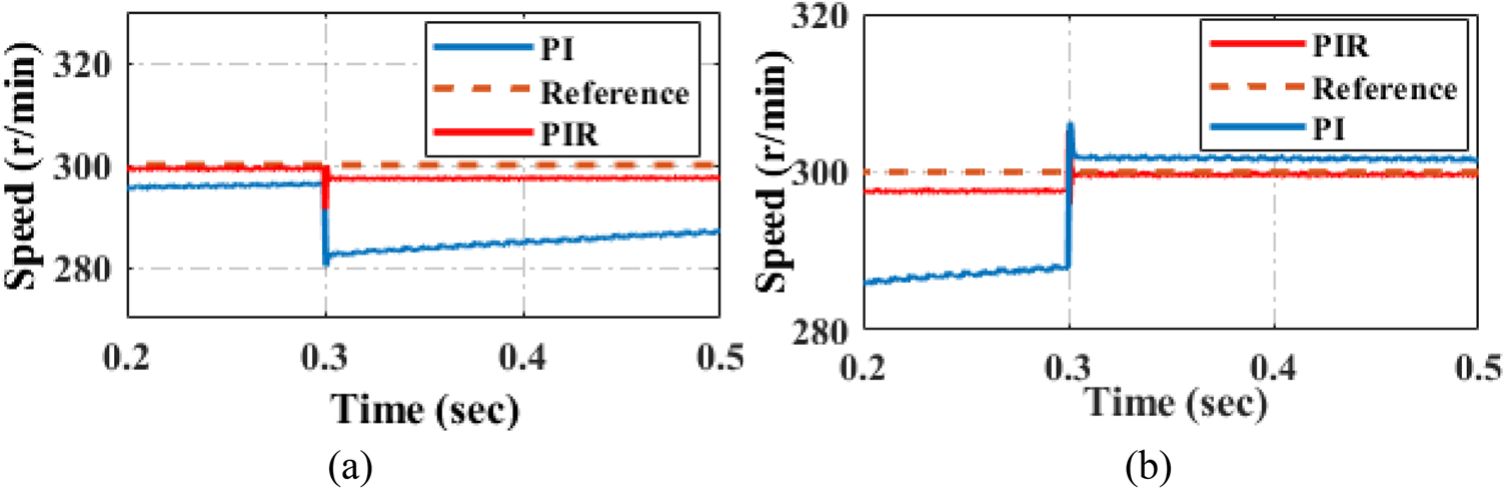
Performance of speed under (**a**) load torque increased (**b**) load torque decreased.

The Fig. 18 illustrates the speed response under the conditions of increasing and decreasing load torque. It shows the system’s behavior during both the load torque increase and decrease phases. From these responses, it is evident that under varying load torque conditions, the proposed controller achieves reference speed more quickly than the PI controller. Hence it can be noted that compared to conventional PI controller, the proposed controller quickly reaches the reference speed when the load variation is occur as shown in Fig. 18a and b respectively. Also, the proposed controller can reduce the overshoot oscillation.

**Fig. 19:**
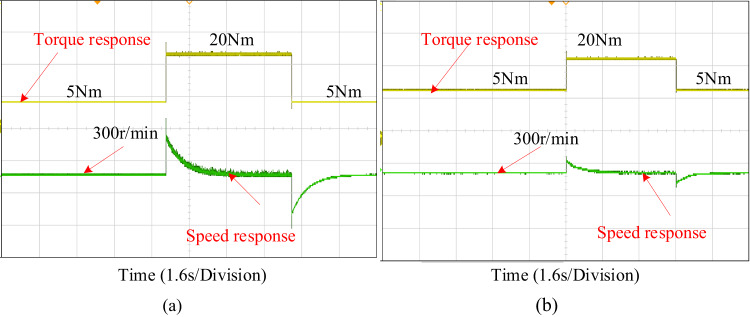
Experimental verification during increased and reduced load torque (**a**) PI control (**b**) PIR control.

Figure 19 displays an experimental comparison of the torque response for the PI and proposed PIR control techniques. For consistency, the same combined simulation test case is used for experimental verification. In the experiment verification, the machine starts with a speed reference of 300r/min and a 5Nm load torque. After a certain period, the load is increased to 20Nm and subsequently decreased back to 5Nm after another interval. Throughout the load variation, the machine maintains a constant speed. The Fig. 19a illustrates the system’s performance under PI controller and the proposed PIR controller is depicted in Fig. 19b. The EV’s output torque stabilizes, and the torque ripple is minimal once it has reached its steady state. The output torque can rapidly follow variations in the load torque when the load reduces, and the adjustment time is the smallest. Additionally, there is very little torque ripple in the steady state, which effectively prevents it. Hence it can be concluded that the proposed control technique can offers the benefits of strong torque dynamic tuning capability and rapid self-recovery compared to conventional PI control system.

**Fig. 20:**
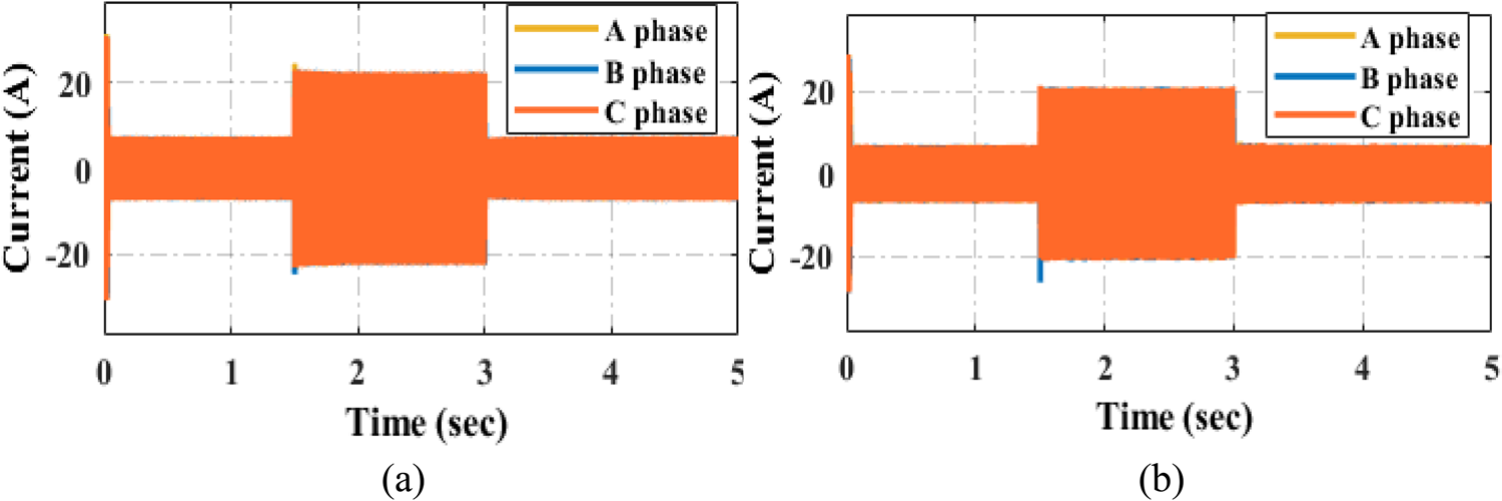
Current response under (**a**) load torque increased (**b**) load torque decreased.

The Fig. 20 illustrates the current tracking response under varying load torque conditions. Figure 20 provides a simulation comparison of the torque response for the PI and proposed PIR control techniques. In this test case, the machine begins operate at a reference speed of 300 r/min with an initial load torque of 5Nm. After a certain interval, the load torque is increased to 20 Nm and subsequently reduced back to 5Nm. Despite these load variations, the motor maintains a constant speed. The Fig. 20a illustrates the current tracking performance under the PI controller, while the proposed PIR controller’s performance is depicted in Fig. 20b. From these responses, it can be observed that under varying load torque conditions, the proposed PIR controller demonstrates faster settling time and superior current tracking performance and the current harmonics of the proposed controller is reduced, hence it should be noted that the efficiency and overall performance is increased as compared to the conventional PI controller.

**Table 5:** Numerical examination of current loop during various load torque conditions.

	Steady state error	Settling time (s)	Overshoot (%)	Current Harmonics (%)
Increased Load (Nm) (5-20Nm)				
PI Controller^46^	0.627	0.05	29	3.01
Proposed PIR Controller	0.09	0.02	2.1	0.45
Decreased Load (Nm) (20-5Nm)				
PI Controller^46^	0.417	0.045	30	2.49
Proposed PIR Controller	0.10	0.09	1.5	0.49

**Fig. 21:**
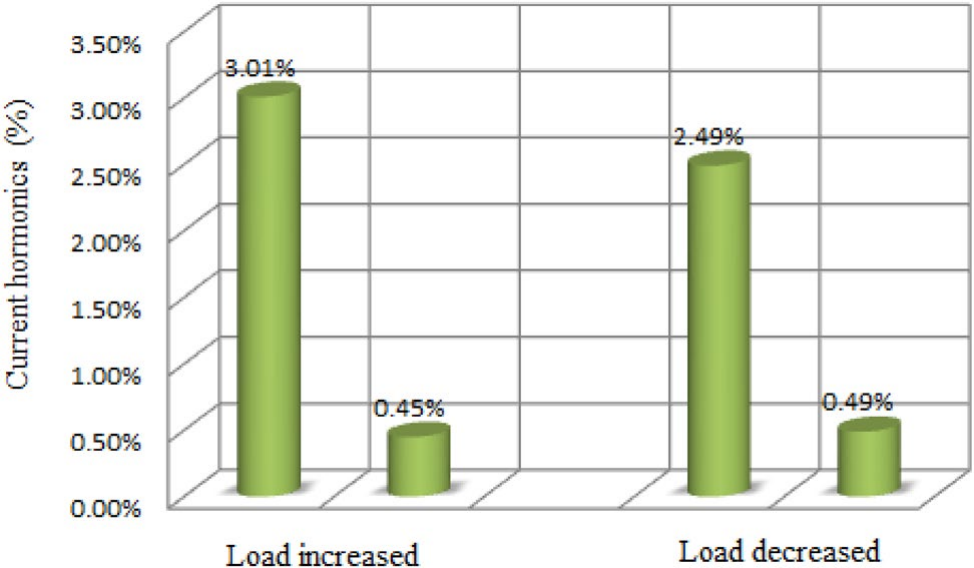
Graphical representation of current harmonics performance comparison.

The quantitative analysis and comparison of steady state error, settling time, overshoot and current harmonics of the load variation is depicted in Table 5 from this table it can be noted that PI control system has steady state error of 0.627, settling time of 0.05 s, overshoot of 29% and current harmonics of 3.01% when the load is increased from 5 to 20Nm. the proposed control system has steady state error of 0.09, settling time of 0.02, overshoot of 2.1% and current harmonics of 0.45%. From this analysis the table can be concluded that compared to PI controller, proposed adaptive PIR controller overall performance is increased, hence, the efficiency of the system also increased. The graphical representations of the current harmonics are illustrated in Fig. 21. From this representation the harmonics of the proposed controller is reduced when both the conditions.

**Fig. 22:**
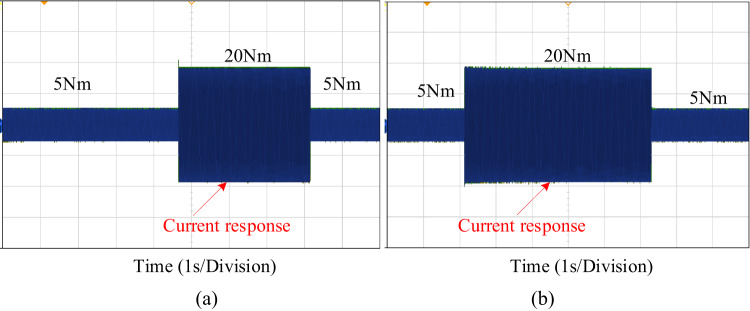
Performance of dynamic load torque under (**a**) PI control (**b**) PIR control.

The Fig. 22 illustrates the experimental verification of the current tracking response under varying load torque conditions. For consistency, the experimental setup replicates the same simulation test case. Figure 22 presents a comparative analysis of the current responses for the PI and proposed PIR control techniques. The overall performance of the PI controller is demonstrated in Fig. 22a, and the PIR controller’s performance is illustrated in Fig. 22b. The results demonstrate that the proposed PIR controller beats the PI controller in all aspects and it can provide superior current tracking and overall performance.

**Fig. 23:**
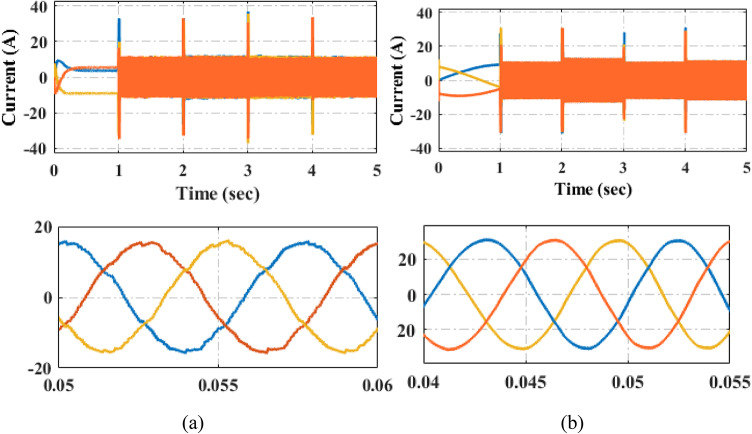
Dynamic current response under (**a**) PI control (**b**) PIR control.

Figure 23 illustrates the current tracking response of the machine at the time of dynamic speed conditions. For verification purpose the scenario used in the test case is same in the test case which is shown in Fig. 13 of speed response. From those test case compared to PI controller the proposed PIR controller can easily swifts the current based the reference speed and it takes less time to reach the steady state condition as depicted in Fig. 23a and b respectively.

**Fig. 24:**
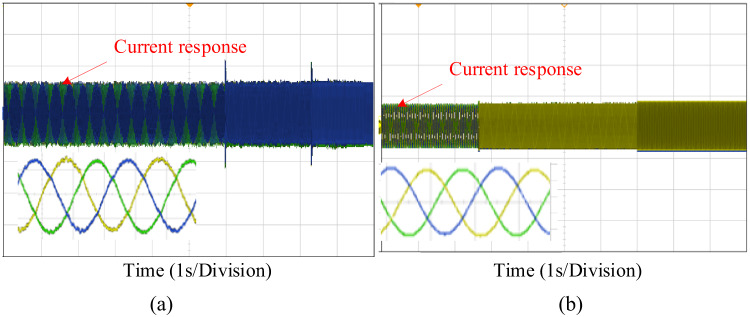
Dynamic current response under (**a**) PI control (**b**) PIR control.

The Fig. 24 illustrates the experimental verification of the current tracking response under dynamic speed conditions. For consistency, the experimental setup replicates the same simulation test case. Those figures present a comparative analysis of the current responses for the PI and proposed PIR control techniques. The performance of the PI controller is demonstrated in Fig. 24a, while the PIR controller’s performance is illustrated in Fig. 24b. The results confirm that the proposed PIR controller outperforms the PI controller in all aspects, providing superior current tracking and overall performance. Hence it can be concluded that the proposed controller can gives best performance outcome in both simulation and experimental verification.

The ac components in $\:{i}_{sq}\:$under PIR control are higher than that of under PI control for the current responses. The speed regulator generates these ac components in order to mitigate speed harmonics. In both control methods, the $\:{i}_{sd}\:$ waveforms are almost the similar outcomes. The speed and current PIR controller have two off-and-on processes of resonant phases, which should be recognized.

**Fig. 25:**
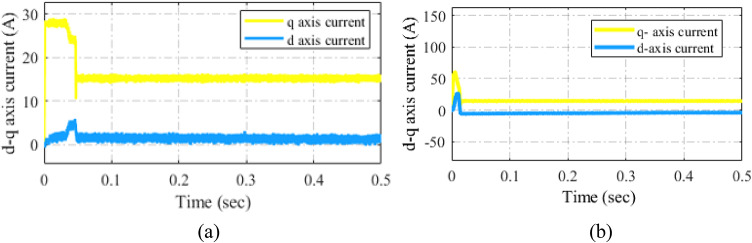
A *d-q* current response under (**a**) PI control (a) PIR control.

The *d-* and *q-* axis speed and current waveforms during conventional PI and PIR current control techniques are displayed in Fig. 25. In this result, the torque load is 15 Nm, and the reference speed is 2000 r/min. Both current control systems use a PI controller in conjunction with a motor speed loop. It is evident that under PI current management, there are periodic harmonics in $\:{i}_{sd}$ reaches 15 A as shown in Fig. 25a and static inaccuracies between $\:{i}_{sq}$and$\:{\:i}_{sqref}$. Nevertheless, $\:{i}_{sq}$ and $\:{i}_{sqref}$ are nearly the same, and when PIR current control is applied, the harmonics in $\:{i}_{sd}$ disappear. This finding suggests that the performance of the current loop is effectively enhanced by the PIR current control approach as illustrated in Fig. 25b.

#### Analysis and verification of parameter variation

The characteristics of resistance, inductance, and other drive system components may alter as a result of EVs operating at high speeds for extended periods of time, which can raise temperatures, or when they are driven in inclement conditions like rain or snow. Furthermore, the controller must be resilient to parameter uncertainty and unmolded dynamics. Because each vehicle’s system specifications differ a bit even among vehicles of the same type, tuning settings can be difficult. As a result, when the parameters modify, the driving system’s performance must be tested. The experimental results are summarized in Tables 6 and 7 which compares how well these control methods function in various operating settings with parameter adjustments.

**Table 6:** Response time analysis of $\:{R}_{s}$

Test Speed	Parameter value	Response time (sec)	
		PI Control^46^	Proposed method
Starting 0 to 2000 r/min	$\:{R}_{s}$ normal	2.08	1.95
	0.034$\:{R}_{s}$(40%)	2.45	2.01
	0.017$\:{R}_{s}$(20%)	2.78	2.33

**Table 7:** Response time analysis of $\:{L}_{s}$

Test speed	Parameter value	Response time (sec)	
		PI Control [46]	Proposed method
Starting 0 to 2000 r/min	$\:{L}_{s}$normal	2.08	1.95
	6,4$\:{L}_{s}$(40%)	3.45	3.01
	3.2$\:{L}_{s}$(20%)	3.79	3.34

Table 6 illustrates the EV’s response time changes as stator resistance increases when it begins at various speeds using the conventional and proposed control techniques. The resistance value of the PI control and proposed control are suggested to substitute 0.017Rs, 0.034Rs, and 0.0852Rs, respectively, for the nominal value of stator resistance in order to replicate the effects of increasing stator resistance by 20%, 40%, and 100%. The inductance value of the two control are suggested to substitute 3.2$\:{L}_{s}$, 6.4$\:{L}_{s}$ and 16$\:{L}_{s}$.The impact of increasing stator inductance by 20%, 40%, and 100%. Experimental evaluation was done on the two control methods’ necessary response time. Table 7 display the findings. Tables 6 and 7 shows that the PI control have longer initial response times compared to proposed control technique when the variation occur in the parameter. Hence the proposed control has obvious robustness when the parameter varies.

#### Effect on computation time

Understanding the computation time of the control algorithm is crucial for the digital controller. The computation times of two different control structures are displayed in Table 8. It is evident that the proposed system computational time is marginally lesser than that of other conventional control technique. This aligns with the design process of the scheme under study in equations (27). Because the system control period is set at 156.4µs, the computational time of the scheme under study only takes up 1/8 of the interval. The investigated scheme can therefore be used in real-time practices. Hence the proposed system calculation time is less as compare to conventional controller.

The computation time of the controller should calculate from controller Eq. (27), the computation time calculation expression yield,

35 $$\:{T}_{Comp}=\:\frac{{N}_{flops}\:}{{f}_{CPU}*flops\:per\:cycle}.$$

Hence $\:{T}_{Comp}$ is computation time, $\:{N}_{flops}$ number of floating point operations, $\:{f}_{CPU}$ is 4.6GHZ

**Table 8:** Computation time comparisons.

Method	Computational time		Computation time	
	Algorithm	Number	MATLAB/Simulink	HIL Setup
PI control [46]	+,− *,/	20 45	22.5 µs	21.05 µs
PIR control	+,− *,/	80 89	17.5 µs	17.1 µs

## Comparative steady and analysis

**Fig. 26:**
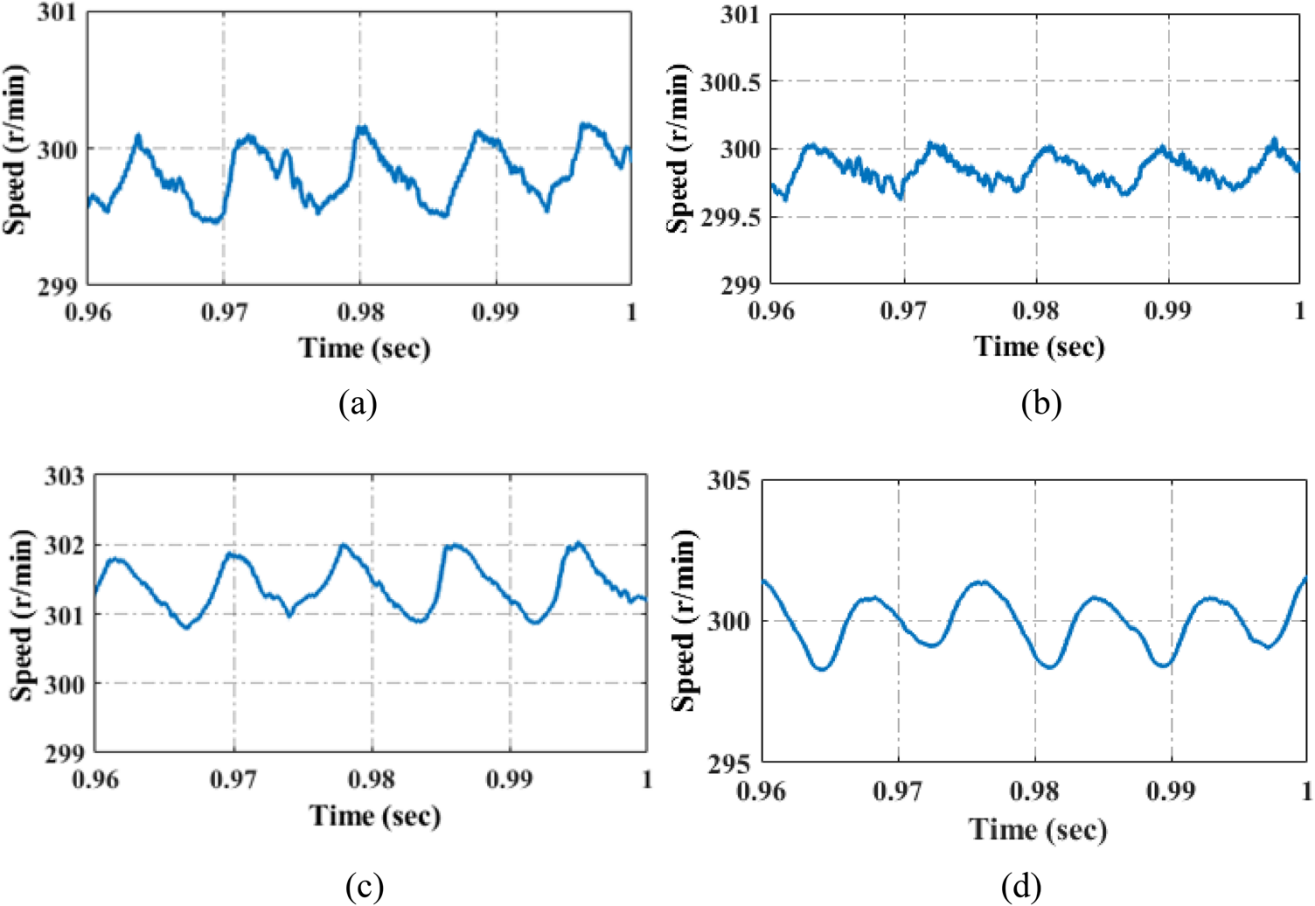
Steady state speed performance under (**a**) PI control (**b**) QPR control (**c**) PR control (**d**) Proposed PIR control.

A comparison of motor speed simulation results using PI control^46^, QPR^30^, PR control^32^, and PIR control is shown in Fig. 26. Table 9 contains the corresponding speed harmonics and THD values results. The simulation uses a machine runs at 300r/min with a load torque of 15Nm. The machine parameters are listed in Table 2. The PR controller has a previous cycle feedback gain of 3, a current cycle feedback gain of 11, and a forgetting factor of 0.07. Figure 26; Table 9 demonstrate that the first, second, and sixth speed ripples are completely eliminated in PIR control and considerably suppressed in PR control compared to QPR and PI controller, the THD analysis of the proposed system and traditional PI, QPR, PR and proposed PIR control is depicted in Table 9. It can be seen that compared to traditional PI, QPR, PR techniques, the proposed PIR THD is reduced hence, the system overall performance and efficiency is increased.

**Table 9:** Comparison analyses of steady state speed harmonics and THD values.

Control method	1st order	2nd order	6th order	12th order	15th order	18th order	THD analysis
PI^46^	2.25%	2.12%	2.05%	1.90%	1.50%	0.50%	3.21%
QPR^30^	2.21	2.02	2.07%	1.87%	1.35%	0.98%	2.55%
PR^32^	2.20%	2.01%	1.99%	1.55%	1.30%	1.08%	1.74%
PIR	0	0	0	0.65%	0.57%	0.41%	0.17%

**Table 10:** Quantitative analysis of steady state error.

Speed (*r*/min)	Steady state error			
	PI control^46^	QPR^30^	PR^32^	PIR
150	3.11	3.09	3.02	2.90
300	3.01	2.87	2.30	2.10
450	2.10	2.01	2.11	1.98
750	1.52	1.20	1.30	1.29
1000	1.39	1.22	1.20	1.00
2000	1.21	1.01	1.00	0.90
3000	1.09	1.08	1.06	0.50
4000	1.08	1.07	1.05	0.49

**Fig. 27:**
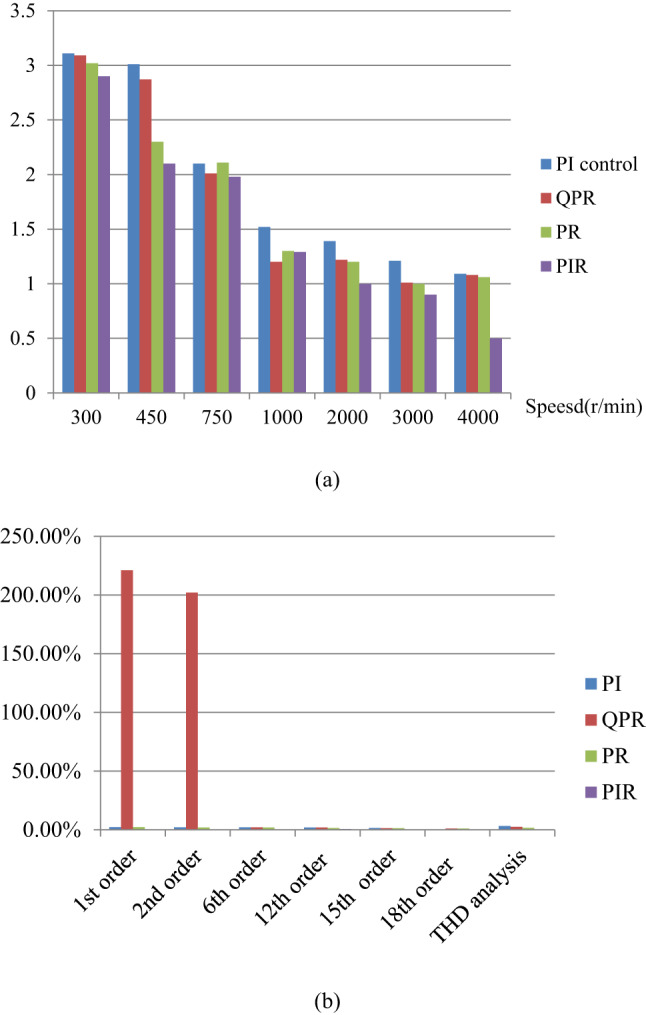
Graphical representation of (**a**) steady state error (**b**) speed harmonics.

In Fig. 27 illustrates the graphical representation comparison over conventional PI, QPR, PR and proposed PIR control techniques at different speed conditions at 15Nm load torque. As shown in Fig. 27a and Table 10 it can be seen that the steady state error of the proposed PIR control is minimum when compared to conventional PI, QPR, PR control techniques. The speed harmonics and THD values of the conventional PI, QPR, PR and the proposed PIR control technique graphical representation is represented in Fig. 27b and Table 9. From this analysis it can be seen that the proposed control speed harmonics and THD values are reduced compare to all the other conventional techniques. This highlight indicates that the proposed PIR control over all performance and efficiency is increased compared to other conventional control technique.

**Fig. 28:**
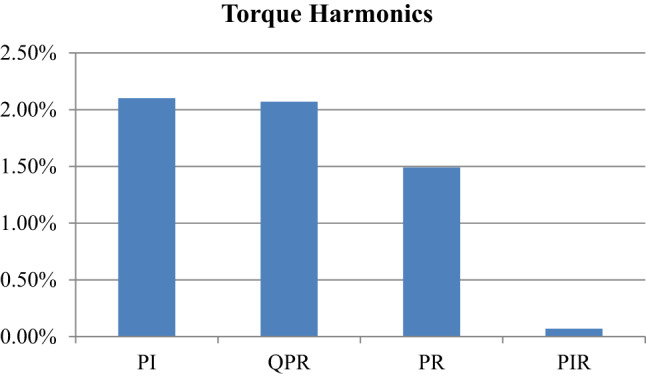
Comparison over the torque harmonics.

The conventional PI, QPR, PR and proposed PIR control comparison of torque harmonics is shown in Fig. 28. From this representation the proposed PIR control torque harmonics is reduced compared to other conventional PI, QPR and PR control technique. Hence, it has reduced vibration and increased overall performance and efficiency of the proposed system.

## Conclusion

The PMSM vector control system’s speed and current loop incorporates an adaptive PIR controller with phase compensation to reduce the first, second and third order harmonic component in steady-state and transient speed due to the non-ideal factor occur in PMSM based EV application. The primary objective of this work is summarized as; (i) to verify the speed- current harmonics caused by the non-ideal factor simulation and experimental results are carried out. (ii) The design of the following parameters ensures that the proposed PIR controller is stable and flexible to changes in speed and motor parameters while maintaining good steady-state and dynamic performance. (iii) The nyquist method, which can provide system stability across the entire speed and current range, limits the stability range of resonant control gain.(iv)A piecewise phase compensation approach is proposed in order to balance the system stability margin and harmonic suppression capability. This method can circumvent the intricate real-time computation of the trigonometric functions for compensation. The proposed PIR control and conventional PI, QPR, PR control schemes are implemented using MATLAB/Simulink and the comparison is made using HIL simulator OPAL-RT OP5700 platform. Hence it can conclude that, the main aim of this proposed work is lowering torque harmonics in PMSMs allows for quieter, smoother operation, significant reductions in motor noise and vibrations, more efficiency, and enhanced overall performance. At a certain frequency value PIR has the good tracking performance. However, the motor speed does not match the reference speed under the dynamic state because there is less system interaction. The challenging part of the proposed control system is tuning process.

In addition with EV application proposed adaptive PIR controller may also use in grid, micro grid and wind energy application.

## Data Availability

All datasets used and/or analysed during the current study available from the corresponding author on reasonable request.
